# BDA-366, a putative Bcl-2 BH4 domain antagonist, induces apoptosis independently of Bcl-2 in a variety of cancer cell models

**DOI:** 10.1038/s41419-020-02944-6

**Published:** 2020-09-17

**Authors:** Tamara Vervloessem, Binu K. Sasi, Elena Xerxa, Spyridoula Karamanou, Justin Kale, Rita M. La Rovere, Supriya Chakraborty, Flore Sneyers, Meike Vogler, Anastassios Economou, Luca Laurenti, David W. Andrews, Dimitar G. Efremov, Geert Bultynck

**Affiliations:** 1 KU Leuven, Laboratory of Molecular and Cellular Signaling, Department of Cellular and Molecular Medicine & Leuven Kanker Instituut (LKI), Leuven, Belgium; 2 Molecular Hematology, International Center for Genetic Engineering & Biotechnology, Trieste, Italy; 3 KU Leuven, Laboratory of Molecular Bacteriology, Rega Institute for Medical Research, Department of Microbiology and Immunology, Leuven, Belgium; 4 Biological Sciences, Sunnybrook Research Institute, Toronto, ON Canada; 5 Institute for Experimental Cancer Research in Pediatrics, Goethe-University, Frankfurt, Germany; 6 Hematology Institute, Catholic University Hospital “A. Gemelli”, Rome, Italy; 7 Sunnybrook Research Institute and Departments of Biochemistry and Medical Biophysics, University of Toronto, Toronto, ON Canada

**Keywords:** Targeted therapies, Target validation, Lymphoma, Chronic lymphocytic leukaemia, Translational research

## Abstract

Several cancer cell types, including chronic lymphocytic leukemia (CLL) and diffuse large B-cell lymphoma (DLBCL) upregulate antiapoptotic Bcl-2 to cope with oncogenic stress. BH3 mimetics targeting Bcl-2’s hydrophobic cleft have been developed, including venetoclax as a promising anticancer precision medicine for treating CLL patients. Recently, BDA-366 was identified as a small molecule BH4-domain antagonist that could kill lung cancer and multiple myeloma cells. BDA-366 was proposed to switch Bcl-2 from an antiapoptotic into a proapoptotic protein, thereby activating Bax and inducing apoptosis. Here, we scrutinized the therapeutic potential and mechanism of action of BDA-366 in CLL and DLBCL. Although BDA-366 displayed selective toxicity against both cell types, the BDA-366-induced cell death did not correlate with Bcl-2-protein levels and also occurred in the absence of Bcl-2. Moreover, although BDA-366 provoked Bax activation, it did neither directly activate Bax nor switch Bcl-2 into a Bax-activating protein in in vitro Bax/liposome assays. Instead, in primary CLL cells and DLBCL cell lines, BDA-366 inhibited the activity of the PI3K/AKT pathway, resulted in Bcl-2 dephosphorylation and reduced Mcl-1-protein levels without affecting the levels of Bcl-2 or Bcl-xL. Hence, our work challenges the current view that BDA-366 is a BH4-domain antagonist of Bcl-2 that turns Bcl-2 into a pro-apoptotic protein. Rather, our results indicate that other mechanisms beyond switching Bcl-2 conformation underlie BDA-366’s cell-death properties that may implicate Mcl-1 downregulation and/or Bcl-2 dephosphorylation.

## Introduction

An important survival strategy of many cancer cells is upregulation of antiapoptotic Bcl-2 proteins^1^. Cancer cells such as chronic lymphocytic leukemia (CLL) and diffuse large B-cell lymphoma (DLBCL) exploit the antiapoptotic effects of Bcl-2 to survive oncogenic stress, though such cells are primed to death^2,3^. Bcl-2 enables cancer cells to evade apoptosis at two levels. At the mitochondria, Bcl-2, mainly via its hydrophobic cleft, sequesters and neutralizes proapoptotic proteins such as Bax, Bak, and BH3-only proteins^1,4^. At the endoplasmic reticulum (ER), Bcl-2, mainly via its BH4 domain, binds and inhibits the inositol 1,4,5-trisphosphate receptor (IP_3_R), preventing proapoptotic Ca^2+^ fluxes from the ER^5–7^. Therefore, much effort has been undertaken in finding a therapeutic strategy to antagonize Bcl-2 with a focus on small molecules that occupy Bcl-2’s hydrophobic cleft^2,8^. BH3 mimetics, such as venetoclax, have been developed to induce apoptosis by targeting Bcl-2’s hydrophobic cleft without directly activating Bax^9^. As cancer cells display high levels of proapoptotic BH3-only proteins due to oncogenic stress, they are sensitive to Bcl-2-antagonizing therapeutics such as venetoclax^3,10^. Currently, a broad scale of on-target BH3 mimetic inhibitors of several anti-apoptotic Bcl-2-family members have been developed^11^.

However, also the targeting of Bcl-2’s BH4 domain recently emerged as a promising strategy to drive cancer cell death^12,13^. One strategy has been to use IP_3_R-derived peptides (such as BIRD-2; Bcl-2/IP_3_ receptor disrupter-2) that represent the binding site for Bcl-2’s BH4 domain^5,14,15^. Such peptides can be used as a decoy for Bcl-2, stripping it from IP_3_R channels and favoring proapoptotic Ca^2+^ fluxes^16,17^. This strategy was effective to kill Bcl-2-dependent cell models, including primary CLL cells^18^, DLBCL cell models^19^, small cell lung cancer (SCLC) cells^20^, and multiple myeloma and follicular lymphoma^21^. The sensitivity of B-cell cancer cells appeared to be dependent on the expression levels of IP_3_R2 channels^19^ in combination with the tonic activation of the B-cell receptor (BCR) provoking Ca^2+^ overload^22^. However, the use of peptides as drug candidates has limitations because of issues with stability and delivery, raising interest in identifying other approaches to target the Bcl-2 BH4 domain.

Recently, a novel small molecule, named BDA-366^23^, has emerged from a small molecule screen as a compound that binds Bcl-2’s BH4 domain with high affinity and selectivity. Binding of BDA-366 to Bcl-2’s BH4 domain was reported to expose its BH3 domain and activate Bax, thus converting Bcl-2 into a proapoptotic protein. As such, BDA-366 provoked cell death in cancer cells expressing high levels of Bcl-2, such as SCLC and non-SCLC (NSCLC) cells. The degree of BDA-366-induced cell death positively correlated with Bcl-2-protein levels, whereby cancer cells expressing the highest level of Bcl-2 displayed the highest sensitivity to BDA-366. Further work revealed that BDA-366 also induces apoptosis in multiple myeloma cell lines and primary samples and delays the growth of xenografted tumors without significant cytotoxic effects on normal hematopoietic cells^24^. This report also suggested that part of the cytotoxic activity of BDA-366 could be due to inhibition of Bcl-2 phosphorylation at Ser70. Thus, BDA-366 emerged as a promising anticancer tool for a variety of cancer types characterized by Bcl-2 overexpression.

CLL and DLBCL are two common B-cell malignancies that frequently display overexpression of Bcl-2, albeit through different mechanisms. In CLL, overexpression of Bcl-2 has been linked to deletion of the microRNAs miR-15 and miR-16^25^, although other mechanisms may also play a role. In DLBCL, overexpression of Bcl-2 most commonly results from the *t*(14,18) translocation, which is detected in ~40% of cases belonging to the GCB-DLBCL subset, or because of BCL2 gene amplification, which occurs in ~30% of ABC DLBCL tumors^25^. In addition, both diseases are characterized by a substantial proportion of cases with a BCR pathway that is chronically activated through either antigen-dependent or antigen-independent mechanisms^26–29^. As such, these two diseases appeared as particularly interesting candidates to evaluate the activity of BDA-366, as this drug would be expected to induce apoptosis both by increasing the amplitude of the Ca^2+^ flux to toxic levels and by inducing the conformational change in Bcl-2 that converts it into a proapoptotic protein. In this study, we evaluated this possibility by testing the activity of BDA-366 against a wide range of DLBCL cell lines and primary CLL samples. We show that BDA-366 induces apoptosis in a substantial proportion of cases, but the mechanism is independent of Bcl-2 and is at least in part related to downregulation of Mcl-1 and inhibition of signaling through the PI3K/AKT pathway.

## Results

### Primary CLL and DLBCL cell lines are sensitive to BDA-366

**Fig. 1:**
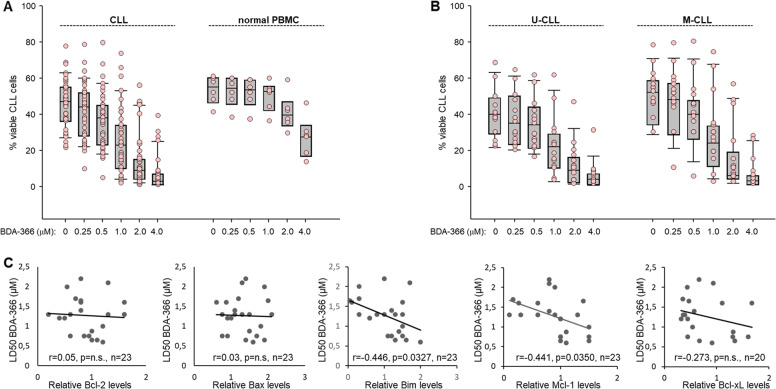
BDA-366 induces apoptosis in primary CLL cells. **a** Cell death was measured in Annexin-V/propidium iodide (PI) stained CLL (*n* = 39) and normal PBMCs (*n* = 6) after 48 h in culture with increasing concentrations of BDA-366. Box plots with data points (pink dots) show the percentage of viable cells. **b** Effects of BDA-366 treatment in U-CLL (*n* = 15) and M-CLL (*n* = 17) cells. **c** Correlation between LD_50_ values and relative levels of Bcl-2, Bax, Bim, Mcl-1, and Bcl-xL. Statistical analysis was performed using the Pearson correlation test.

To investigate whether BDA-366 can kill CLL cells with higher efficiency than normal cells, we analyzed the viability of CLL (*n* = 39) and normal peripheral blood mononuclear cells (PBMCs) (*n* = 6) incubated with BDA-366 for 48 h (Fig. 1a). Annexin V-FITC/PI analysis showed that BDA-366 is significantly more toxic against CLL (LD_50_ = 1.11 ± 0.46 µM) than normal PBMCs (LD_50_ = 2.03 ± 0.31 µM, *P* < 0.001). However, considerable variability was noted in the sensitivity of the different CLL samples towards BDA-366, with approximately 50% of the cases demonstrating LD_50_ values in the 500 nanomolar range and the remaining cases displaying sensitivity similar to normal PBMC. To investigate the reasons for this variability, we correlated LD_50_ values with IGHV mutation status and expression of various Bcl-2 family members, including Bcl-2, Bcl-xL, Mcl-1, Bim, and Bax (Fig. 1b, c and Supplementary Fig. 1). Interestingly, the only positive finding from this analysis was the inverse correlation between LD_50_ and expression of Bim and Mcl-1, whereas no correlation was observed with IGHV mutation status and expression of Bcl-2, Bcl-xL, or Bax.

**Fig. 2:**
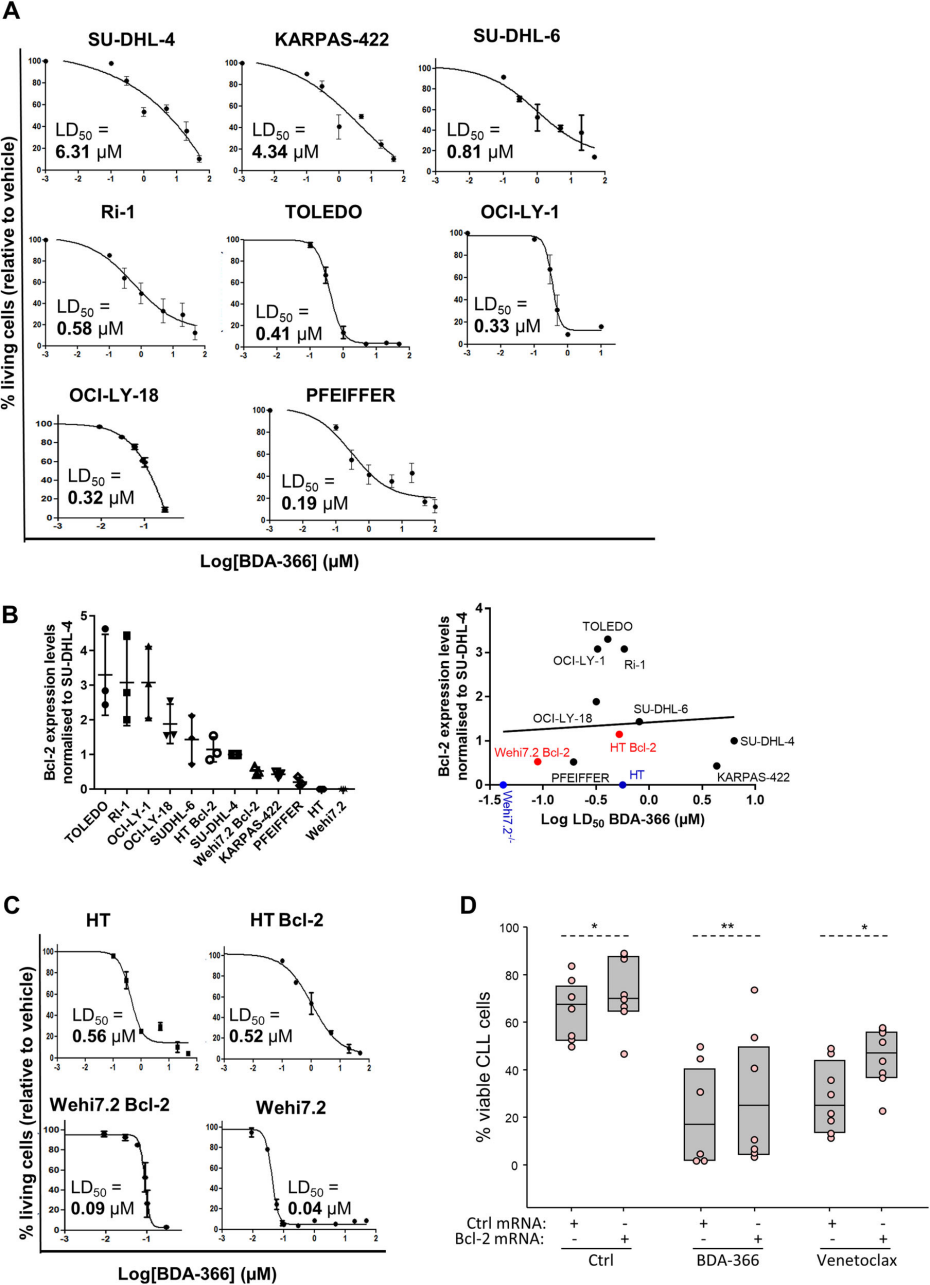
Induction of apoptosis by BDA-366 in DLBCL. **a** Concentration-response curves of the DLBCL cell lines SU-DHL-4, KARPAS-422, SU-DHL-6, Ri-1, TOLEDO, OCI-LY-1, OCI-LY-18, and PFEIFFER treated with increasing concentrations of BDA-366. After 24 h the level of apoptosis was measured using flow cytometeric analysis in Annexin V-FITC/7-AAD stained cells. Data represented here are averages ± SEM of at least three independent experiment (*N* > 3). **b** Quantification of the expression levels of Bcl-2 in several cell lines (right) normalized to SU-DHL-4 (left). Correlation plot of the LD_50_ values for BDA-366-induced cell death and different Bcl-2-family protein expression levels in distinct cell lines obtained via linear regression analysis (right). Data are presented as average ± SD of three independent experiments (*N* = 3). **c** Concentration-response curves of two DLBCL cell lines and two murine thymocytes respectively: HT, HT overexpressing Bcl-2 (HT Bcl-2), Wehi7.2 overexpressing (Wehi7.2 Bcl-2) and Wehi7.2. Cell lines were treated with increasing concentrations of BDA-366. After 24 h the level of apoptosis was measured using flow cytometric analysis in Annexin V-FITC/7-AAD stained cells. Data represented here are averages ± SEM of at least three independent experiment (*N* > 3). **d** Overexpression of Bcl-2 in primary human CLL cells by Bcl-2 mRNA transfection increases resistance to BDA-366 and venetoclax. Primary human CLL cells (*n* = 6) were transfected with control (Ctrl) or Bcl-2 mRNA, left in culture for 3 h to achieve maximal Bcl-2-protein expression, and were then exposed to BDA-366 (1 μM) or venetoclax (4 nM). The viability of the leukemic cells was determined after 20 h by Annexin V/PI staining. Statistical analysis was done with the paired *t* test for the comparison of the control and the venetoclax-treated cells, whereas because of non-normal distribution the Wilcoxon Signed Rank test was applied for the comparison of the BDA-366-treated cells.

To further examine this heterogeneity, we studied a collection of Bcl-2-dependent DLBCL cell lines. The different cell lines were treated with increasing concentrations of BDA-366 for 24 h to examine the potential of BDA-366 to induce apoptosis (Fig. 2a). BDA-366 caused cell death with LD_50_ values ranging from nanomolar (PFEIFFER, 0.19 µM; OCI-LY-18, 0.32 µM; OCI-LY-1, 0.33 µM; TOLEDO, 0.41 µM; Ri-1, 0.58 µM; SU-DHL-6, 0.81 µM) till micromolar range (KARPAS-422, 4.34 µM; SU-DHL-4, 6.31 µM) (Fig. 2a). To substantiate that cell death occurred via apoptosis, we quantified the caspase-3 activity using flow cytometry analysis of NucView caspase-3-stained cells (Supplementary Fig. 2A). Caspase-3 activity was induced with relative potencies that paralleled the data from the AnnexinV-FITC/7-AAD analysis for all cell lines except for KARPAS-422, which appeared more sensitive with this assay. That notwithstanding, both the Annexin V staining and the caspase-3 analysis demonstrated that BDA-366 kills DLBCL cells through apoptosis induction.

Subsequently, we examined the importance of Bcl-2 for the BDA-366-induced death of DLBCL cells. As in the case of the previous experiments with primary CLL cells, the Bcl-2-protein levels of our DLBCL collection were analyzed by immunoblotting (Supplementary Fig. 2B), normalized to the Bcl-2-protein level in SU-DHL-4 (Fig. 2b, left panel), and correlated with the LD_50_ values (Fig. 2b, right panel). Consistent with the findings from the experiments with CLL cells, sensitivity towards BDA-366 did not correlate with Bcl-2-expression levels. To underscore these findings, we used the DLBCL cell line HT and the T cell line Wehi7.2, which both have very low endogenous Bcl-2 levels (blue dots in Fig. 2b). These cells should be resistant to BDA-366 if this compound causes cell death by triggering a proapoptotic conformational switch of the Bcl-2 protein. However, both cell lines were very sensitive to BDA-366, suggesting that BDA-366-induced cell death is independent of Bcl-2 (Fig. 2c). Consistently, HT and Wehi7.2 cells stably transfected with Bcl-2 did not become more sensitive to BDA-366 compared to their wild-type counterparts. Moreover, transient overexpression of Bcl-2 in primary human CLL cells resulted in increased resistance to both BDA-366 and venetoclax, further suggesting that BDA-366 does not induce apoptosis by converting Bcl-2 into a proapoptotic protein (Fig. 2d and Supplementary Fig. 3A, B).

### BDA-366 results in Bax activation in living cells

**Fig. 3:**
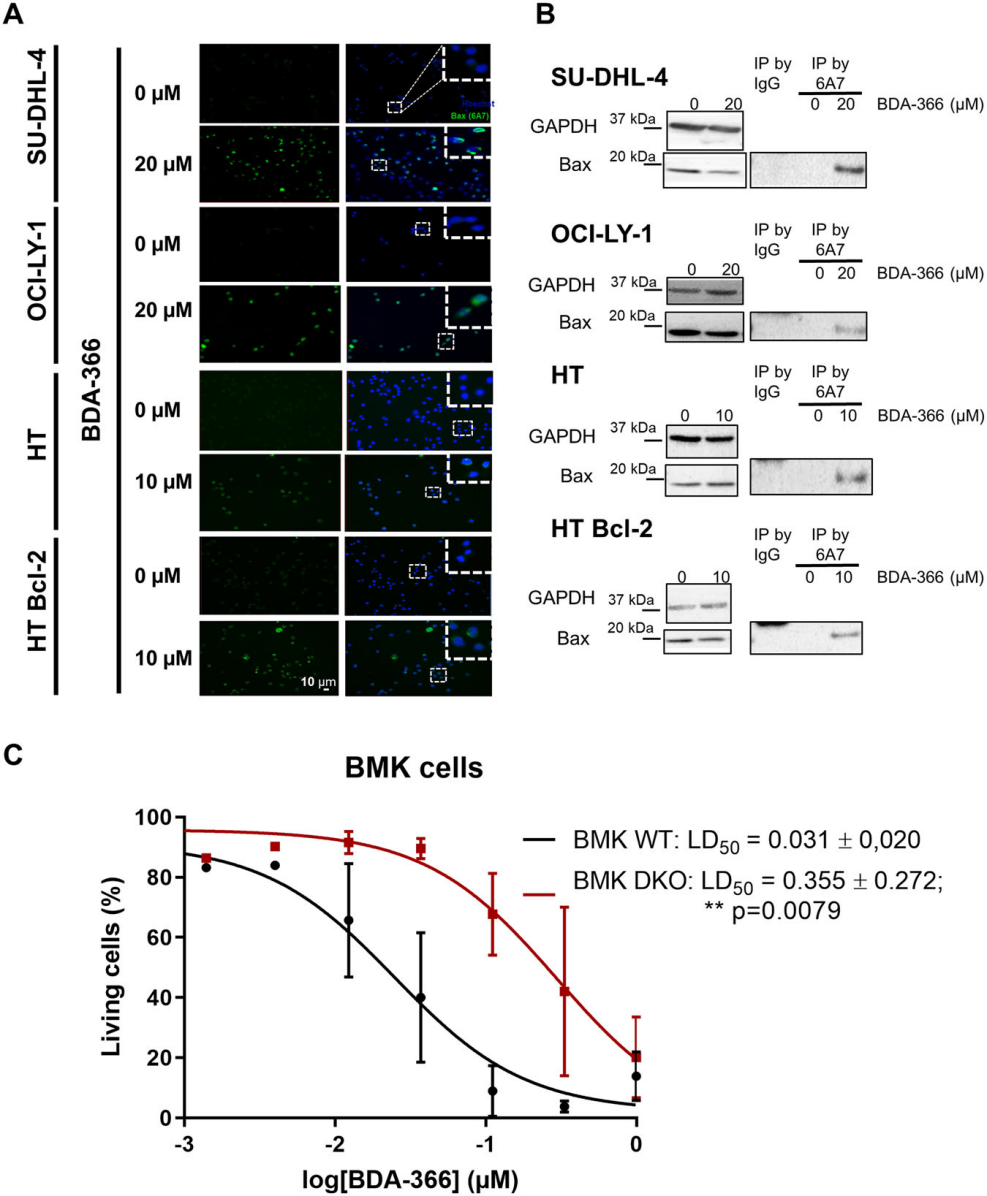
BDA-366 causes Bax activation in different DLBCL cell lines. **a** Representative immunocytochemistry images demonstrating the activation of Bax in DLBCL cells 6 h post incubation with BDA-366. Cells were stained with an antibody that specifically detects the active form of Bax (anti Bax-6A7 antibody). The apoptotic nuclei were stained using Hoechst 33342. Representative images from *N* = 3 independent experiments. **b** Representative co-immunoprecipitation experiment to investigate the activation of Bax upon BDA-366 treatment. The binding to mouse IgG is used as a negative control. A 15 µg of total lysate was used as input. Data representative of *N* = 2 independent experiments. **c** BDA-366 induces Bax-mediated apoptosis. Concentration–response curves of BDA-366 in wild-type (WT) and Bax/Bak double knockout (DKO) Baby-Mouse kidney (BMK) cells. These cell lines were treated with increasing concentrations of BDA-366 after 24 h and the level of apoptosis was measured by Annexin V-Alexa fluor 488 and TMRE staining. Data are presented as average ± SD of *N* = 5 independent experiments and statistical analysis using the Mann–Whitney test.

Next, we wondered whether BDA-366 could activate Bax and if so, whether this occurred via Bcl-2. We therefore focused on 4 cell models, including two Bcl-2-dependent DLBCL cell lines (SU-DHL-4 and OCI-LY-1), one DLBCL cell line lacking Bcl-2 (HT) and HT cells overexpressing Bcl-2. Bax activation was monitored by using the anti-Bax 6A7 antibody, which specifically binds to the active form of Bax. This antibody was used for immunofluorescent staining, where Bax activation correlates with the formation of perinuclear punctae, and in immunoprecipitation approaches, where Bax activation correlates with increased Bax levels in the immunoprecipitate. Importantly, all four cell models, including HT cells that lack endogenous Bcl-2, displayed a robust activation of Bax in response to BDA-366 in nearly all cells (>90% of the cells). These data further suggest that BDA-366 acts independently of Bcl-2 (Fig. 3a, b).

Next, we wished to assess whether BDA-366-induced cell death was dependent on Bax/Bak, and thus canonical apoptosis. Therefore, we compared wild-type versus Bax/Bak double knockout (DKO) baby-mouse kidney (BMK) cells (Fig. 3c). DKO BMK cells (LD_50_ = 0.355 ± 0.272 µM; *N* = 5) were more resistant toward BDA-366 treatment than wild-type BMK cells (LD_50_ = 0.031 ± 0.02 µM; *N* = 5), whereby the LD50 values were significantly different using the Mann–Whitney test (*p* < 0.01). Hence, these data suggest that BDA-366-induced cell death is partially dependent on Bax/Bak or at least is facilitated by Bax/Bak. However, Bax/Bak-independent mechanisms may also contribute, particularly at higher BDA-366 concentrations.

### BDA-366 does neither directly activate Bax nor sensitize Bax toward cBid nor turns Bcl-2 into a Bax-activating protein

**Fig. 4:**
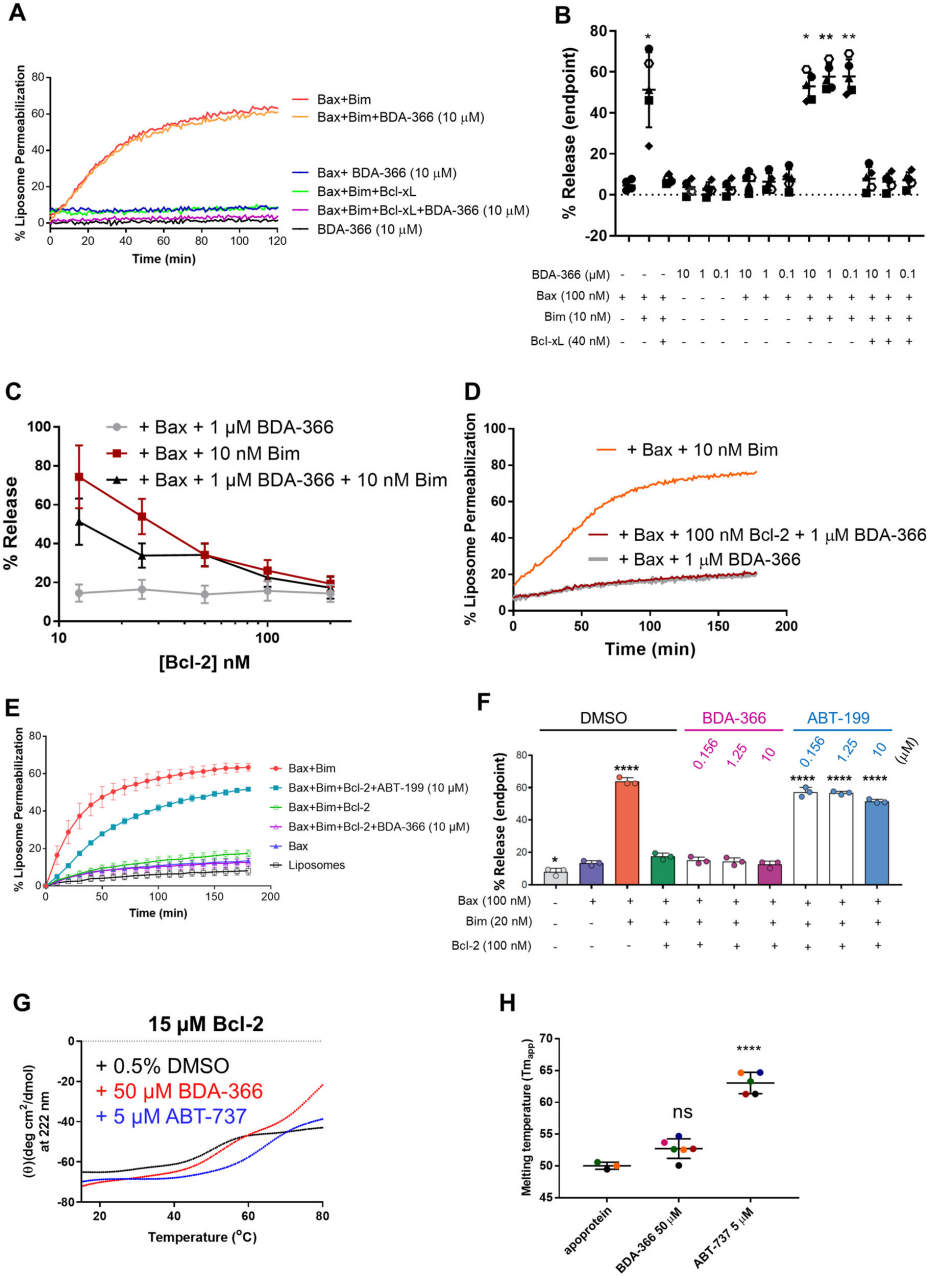
BDA-366 does neither directly activate Bax nor indirectly via Bcl-2. **a** Representative plot of the liposome permeabilisation assay with BDA-366 and different Bcl-2-family members. Liposomes (0.04 mg/mL) encapsulating ANTS and DPX were incubated with BDA-366 (0.1–10 µM), Bim (10 nM) and Bax (100 nM) in the presence or absence of Bcl-xL (40 nM). Liposomes incubated with Bax (100 nM) and Bim (10 nM) were used as positive control, while DMSO (1%) served as the vehicle control. Fluorescent intensity of ANTS (excitation = 355 nm and emission = 520 nm) was measured during 120 min, whereas Bax (100 nM) was added at *t*_0_. **b** Results obtained from endpoint measurements are presented as mean ± SD of five independent experiments. Significance, compared to control condition with only Bax, was determined using a one-way Anova with post hoc Dunn’s multiple comparisons test. **c** Analysis of permeabilisation of liposomes incubated with 10 nM Bim and 100 nM Bax and increasing concentrations of Bcl-2 (*N* = 3). **d** Representative plot of the liposome permeabilisation assay with BDA-366 and Bcl-2. Liposomes encapsulating ANTS and DPX and were incubated with 1 µM BDA-366; 100 nM of Bcl-2 and Bax (100 nM). Fluorescent intensity of ANTS (excitation = 355 nm and emission = 520 nm) was measured during 120 min. Liposomes incubated with Bax (100 nM) and Bim (10 nM) were used as positive control. **e** Similar experiment as in Panel A using varying concentration of BDA-366 or ABT-199 (ranging from 0 to 10 μM), Bcl-2^ΔTMD^ (100 nM), Bax (100 nM), and Bim (20 nM). Data are presented as averages ± SD of three independent experiments at 10 min intervals for 180 min. **f** Results obtained from endpoint measurements are presented as mean ± SD of three independent experiments. Significance, compared to control condition with only Bax, was determined using a one-way Anova with post hoc Dunnett’s multiple comparisons test. **g** Thermal denaturation curves of Bcl-2 (15–85 °C) were obtained by monitoring ellipticity at 222 nm, by far-UV CD, while heating the protein samples [15 µM; 250 µL; 5 mM MOPS, pH: 7.5; 5 mM NaCl; 0.5% DMSO; BDA-366 (50 µM) or ABT-737 (5 µM) as indicated] at 1 °C/min. A representative experiment is shown. **h** Quantitative analysis of Tm_app_ of purified Bcl-2, in the presence or not of drugs (as indicated), obtained from experiments similar to those presented in (**g**). Data presented here are averages ± SD of independent experiments (*N* > 3). Statistical significance was determined with a one-way ANOVA with a Tukey’s multiple comparisons test comparing apoprotein with BDA-366 or ABT-737.

Next, we wished to examine the direct impact of BDA-366 on Bax-pore formation. We therefore used an in vitro Bax-pore formation assay using liposomes and purified Bax. In these experiments, Bax activation leads to liposome permeabilization, resulting in the release of a quencher and an increase in the fluorescence of the ANTS probe. We used a broad range of BDA-366 concentrations (from 0.1 to 10 μM). We first tested whether BDA-366 could permeabilize liposomes by itself, but this was not the case. When adding purified Bax alone, BDA-366 did not provoke liposome permeabilization, indicating that the drug did not directly activate Bax (Fig. 4a and Supplementary Fig. 4A). Incubation of Bax with the activator BH3-only protein Bim resulted in liposome permeabilization that was inhibited when Bcl-xL was added. Addition of BDA-366 did neither enhance Bim-mediated activation of Bax, nor affected Bcl-xL-mediated inhibition of Bax activation. Similar results were observed when cBid was used as the Bax activator (Supplementary Fig. 4C). Next, we performed the key experiment to determine whether or not BDA-366 could turn Bcl-2 into a pro-apoptotic protein that activates Bax by using purified Bcl-2. First, we validated that our purified Bcl-2 protein displayed antiapoptotic activity. Indeed, Bcl-2 prevented Bim-induced Bax-pore formation and thus liposome permeabilization in a concentration-dependent manner (Fig. 4c). Different concentrations of Bcl-2 (ranging from 25 to 200 nM; data shown for 100 nM Bcl-2 in Fig. 4d) were screened to assess whether BDA-366 (1 μM) could activate pore formation by Bax (100 nM) through Bcl-2 (Fig. 4d). We also tested whether BDA-366 could directly activate Bax in the absence of Bcl-2. The BDA-366 concentration was always in excess of the Bcl-2 and Bax-protein concentrations. Bim was included as a positive control for Bax-mediated liposome permeabilization. Again, BDA-366 did neither directly activate Bax in the absence of Bcl-2 nor indirectly via Bcl-2 (Fig. 4d). These data indicate that BDA-366 does not switch Bcl-2 into a pro-apoptotic protein that activates Bax. Finally, we benchmarked BDA-366 against venetoclax (ABT-199), a *bona fide*, selective, BH3 mimetic inhibitor of Bcl-2. For this, we assessed the impact of a broad range of concentrations of BDA-366 or ABT-199 (from 0 to 10 μM) on Bcl-2-mediated inhibition of Bim-induced Bax-pore formation (Fig. 4e, f). Importantly, while ABT-199 efficiently alleviated the inhibition of Bcl-2 on Bim-induced Bax-pore formation, BDA-366 up to 10 μM was not able to do this. This further demonstrates that, in contrast to ABT-199, BDA-366 does not inhibit Bcl-2’s antiapoptotic action.

To further validate whether BDA-366 could impact Bcl-2 structure, we established Bcl-2 melting temperature curves by measuring the CD spectrum at increasing temperatures (Fig. 4g, h). This is a very sensitive manner to study the Bcl-2 conformation. Drugs that impact the conformation would alter the melting temperature. We used 50 μM BDA-366 to have excess drug compared to Bcl-2. However, establishing melting curves for Bcl-2 + DMSO or Bcl-2 + BDA-366 (50 μM) did not provoke substantial changes in the apparent melting temperature, suggesting that BDA-366 did not trigger major changes in the Bcl-2 conformation (Fig. 4e, f). In contrast, ABT-737, a BH3 mimetic drug that binds to Bcl-2 with high affinity, significantly stabilized Bcl-2’s structure, even though this drug was applied at 5 μM, i.e., 10-fold lower concentrations than BDA-366 (Fig. 4e, f). This experiment underpins our technical capacity to measure Bcl-2 stability and demonstrates that although BDA-366 was proposed to provoke a major conformational change, it only had a very limited impact on Bcl-2’s structure.

### BDA-366 does not provoke cytosolic calcium response in DLBCL or CLL primary cells

**Fig. 5:**
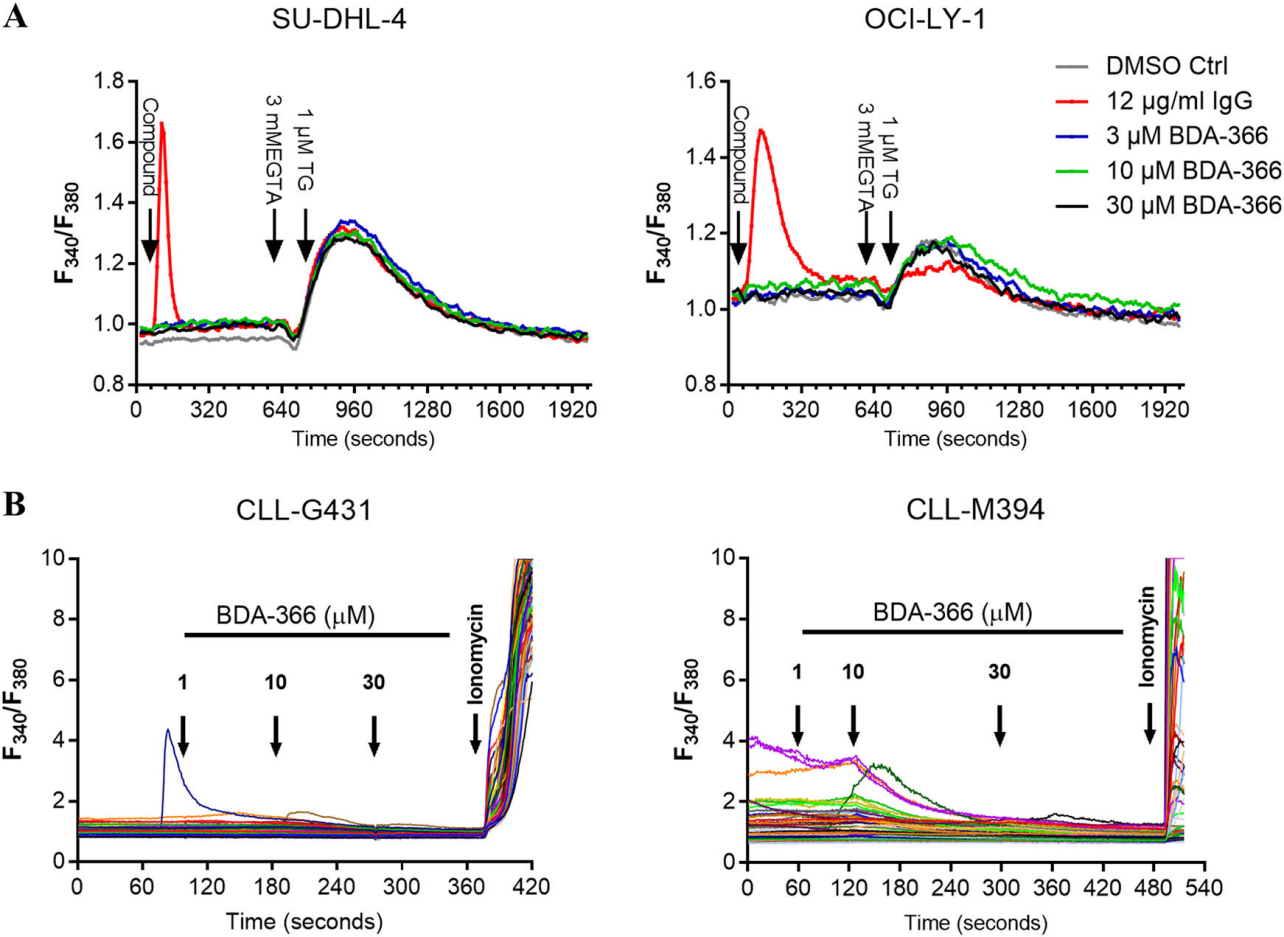
Acute addition of BDA-366 does not increase intracellular calcium concentration. **a** Cytosolic Ca^2+^ measurement in Fura-2 AM loaded SU-DHL-4 (left) and OCI-LY-1 (right) cells. The compound was added after 60 s (DMSO as control, BDA-366 or IgG/IgM) at the indicated concentrations. Each curve represents the F340/F380 ratio as function of time for cell populations from three separate wells, which were considered as technical replicates. One representative experiment (out of six or four independent experiments, presented in Supplementary Fig. 5) is shown in panel (**a**). **b** Single cells cytosolic Ca^2+^ measurement in Fura-2 AM loaded primary CLL samples. Each curve represents the F340/F380 ratio as a function of time for an individual cell. In each experiment, at least 20 cells were measured. One representative experiment (out of three independent experiments) is shown in panel (**b**). Data are representative of three independent measurements per patient sample (CLL-G431 and CLL-M394).

Next, we wished to investigate whether BDA-366 could dysregulate Ca^2+^ signaling. Therefore, the Bcl-2/BCR-dependent cell lines SU-DHL-4 and OCI-LY-1 were loaded with FURA-2 AM and intracellular Ca^2+^ response was monitored upon the addition of BDA-366 (3–30 µM) (Fig. 5a and Supplementary Fig. 5). For these acute treatments, relatively high concentrations of BDA-366 were applied to maximize the chance of observing changes in [Ca^2+^]. Acute addition of different concentrations of BDA-366 did not lead to any cytosolic Ca^2+^ rise in either of the cell lines, while both cell lines displayed a Ca^2+^ response upon stimulation of the BCR with an anti-Ig antibody (Fig. 5a). Also, assessment of the ER Ca^2+^ store content by using an EGTA/thapsigargin approach indicated that BDA-366 did not provoke depletion of the ER Ca^2+^ stores (Fig. 5a). Moreover, we observed similar results in primary CLL cells (Fig. 5b). Upon the addition of increasing doses of BDA-366 (1–30 µM), we did not observe any changes in cytosolic calcium level (Fig. 5b). At the end of the experiment, ionomycin (10 µM) was applied as a positive control to induce Ca^2+^ release from the internal stores. Altogether, these results indicate that BDA-366 does neither trigger cytosolic Ca^2+^ mobilization by itself nor modulate the ER Ca^2+^ content in Bcl-2-dependent cancer models.

### BDA-366 treatment decreases Mcl-1 expression

**Fig. 6:**
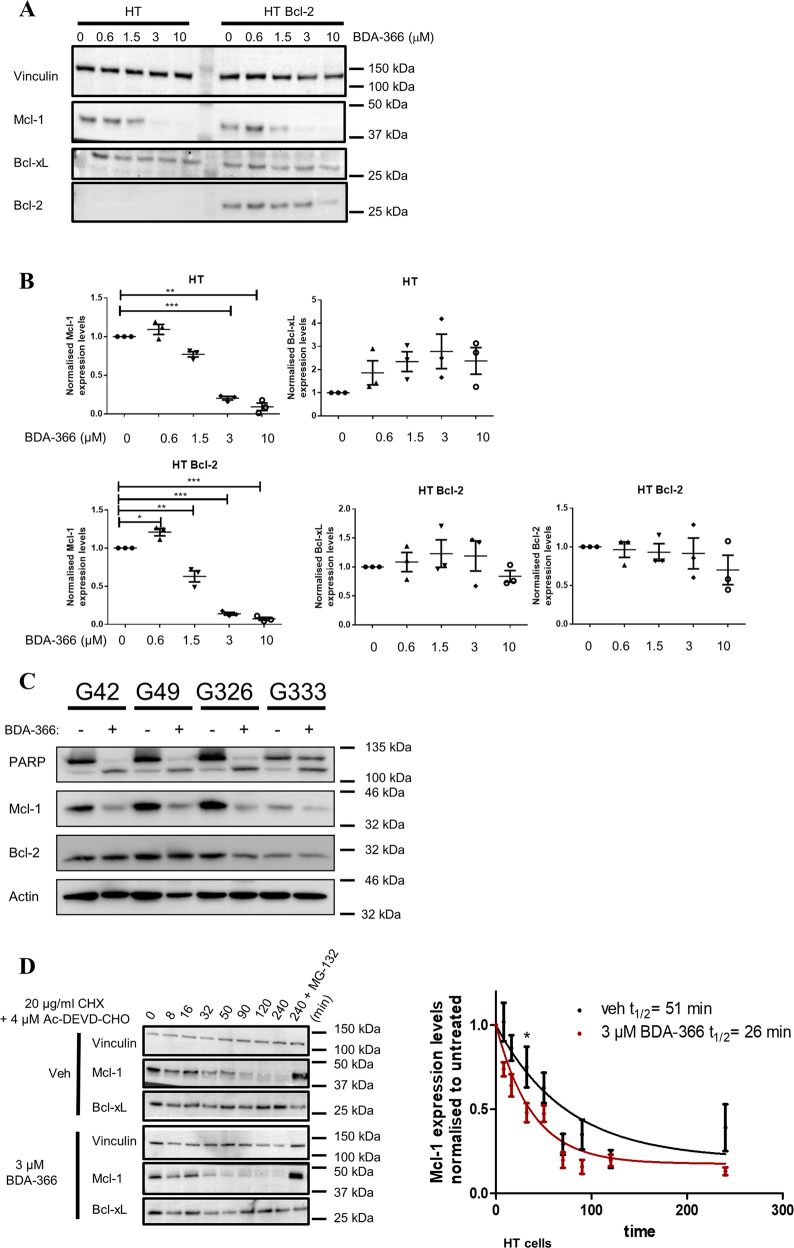
BDA-366 augments Mcl-1 turnover in a Bcl-2-independent manner. **a** Representative western blot of different Bcl-2 family members after BDA-366 treatment for 6 h in HT and HT overexpressing Bcl-2 positive (HT Bcl-2) DLBCL cells. Vinculin was used as a loading control. **b** Quantitative analysis of the different Bcl-2 family members (Mcl-1, Bcl-2, and Bcl-xL). Data are presented as average ± SD of three independent experiment (*N* = 3) with *p < 0.05, **p < 0.01, ***p < 0.001 obtained via a two-tailed paired *t* test. **c** Immunoblotting analysis of PARP cleavage (indicator for apoptosis), Mcl-1 and Bcl-2 levels after 48 h treatment of CLL cells with 2 µM BDA-366. Actin was used as loading control. **d** HT cells were incubated for different time points with 3 µM BDA-366 in combination with 20 µg/ml CHX and the capsase-3 inhibitor (Ac-DEVD-CHO, 4 µM) and subjected to Mcl-1 immunoblotting. The 4-hours time point was analyzed with or without proteasome inhibitor (MG-132, 20 µM). The protein concentrations relative to time 0 were fitted using a one-phase exponential-decay to calculate the half life (*t*_1/2_). Significance was measured via a paired t-test comparing vehicle versus BDA-366 treatment at each timepoint.

Next, we wished to examine the effect of BDA-366 treatment on the expression levels of different antiapoptotic Bcl-2-family members. Both HT and HT Bcl-2 cells were treated with increasing concentrations of BDA-366 (0.6–10 µM) for 6 h, a relatively early time point, and expression levels of antiapoptotic Bcl-2-family members were analyzed (Fig. 6a, b). These BDA-366 concentrations were used as they are near or higher than the LD_50_ concentration observed in the cell death experiments. A significant decrease in the steady state Mcl-1 expression appeared at concentrations of 3 µM and higher in HT and HT Bcl-2 cells while no effect on the expression levels of Bcl-2 or Bcl-xL was observed. Moreover, a significant reduction in Mcl-1-expression levels was also observed in CLL cells upon BDA-366 treatment while the Bcl-2-expression levels remained the same (Fig. 6c). Accordingly, Mcl-1-protein turn-over was examined in DLBCL cells in the absence or presence of BDA-366, whereby protein synthesis was inhibited using cycloheximide (CHX, 20 µg/ml) in the HT cells. To ascertain that downregulation of Mcl-1 is not a consequence of reduced cell viability, we also added a caspase-3 inhibitor in this experiment (Ac-DEVD-CHO, 4 µM) (Fig. 6d). Via immunoblotting the expression levels of Bcl-xL and Mcl-1 were monitored. BDA-366 significantly enhanced the turnover of the Mcl-1 protein, resulting in a decrease in the half-life values from 51 to 26 min. Moreover, proteasomal inhibition with MG-132 (20 µM, 4 h) restored Mcl-1-protein levels (Fig. 6d). Consequently, these data suggest that BDA-366 affects Mcl-1 expression in a Bcl-2-independent manner.

**Fig. 7:**
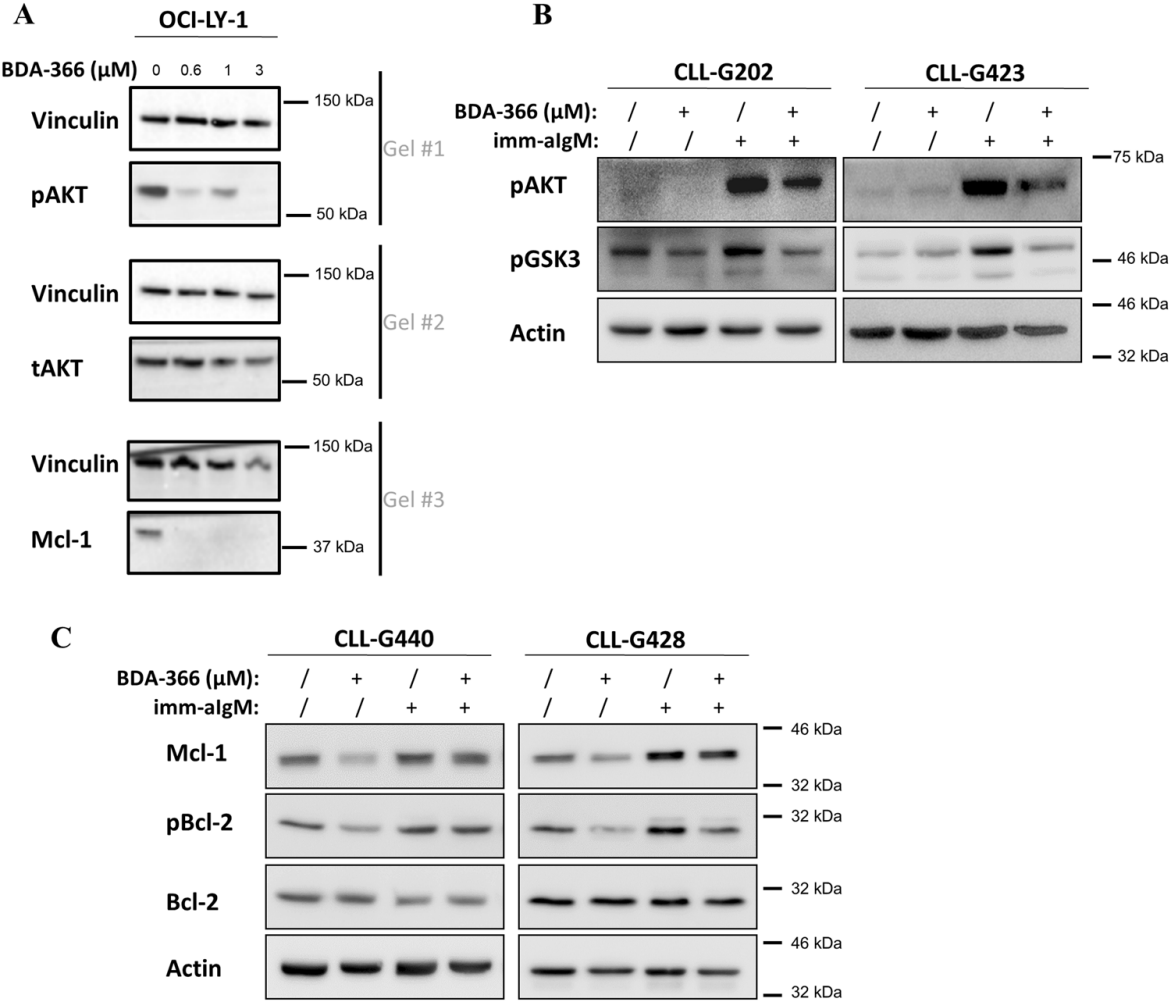
BDA-366 decreases the phosphorylation of AKT and Bcl-2. **a** Representative western blots of pAKT, tAKT, and Mcl-1 in OCI-LY-1 cells (LD_50_ for BDA-366: 0.32 μM) treated for 6 h with 0-0.6-1-3 µM BDA-366. Vinculin was used as a loading control. One representative experiment out of 3 performed is shown. **b** BDA-366 inhibits BCR-induced AKT and GSK3 phosphorylation in primary CLL cells. Cells were pretreated with BDA-366 (2 μM) for 90 min prior to being stimulated with immobilized anti-IgM (imm-aIgM) for 30 min (2 × 10^7^ beads coated with 20 μg goat anti-human IgM antibody per 1 × 10^7^ cells)^34^. **c** BDA-366 inhibits BCR-induced Mcl-1 upregulation and Bcl-2 phosphorylation in primary CLL cells. Cells were pretreated with BDA-366 (2 μM) as above and then stimulated for 24 h with imm-aIgM prior to harvesting for immunoblotting analysis.

Importantly, BDA-366 is a member of the class of anthraquinone derivatives (PubChem CID # 91826545; https://pubchem.ncbi.nlm.nih.gov/compound/bda-366), which are known inhibitors of the PI3K/AKT pathway^30,31^. This pathway via mTOR signaling regulates Mcl-1 translation and via GSK3 regulates Mcl-1 degradation^27,32^. Thus, the AKT-expression and activation were assessed in OCI-LY1 cells exposed to increasing concentration of BDA-366 (0.6–10 µM) for 6 h (Fig. 7a). A decrease of phospho-AKT (pAKT) appeared already with low doses of BDA-366 (0.6 and 1 µM). The level of total AKT (tAKT) was not affected by BDA-366, whereas downregulation of Mcl-1 was observed at the same concentrations that inhibited phospho-AKT.

To further validate these findings, we investigated whether BDA-366 will inhibit phosphorylation of AKT and GSK3 in BCR-stimulated primary CLL cells (Fig. 7b). Pretreatment of CLL cells with BDA-366 resulted in a substantial reduction of anti-IgM-induced AKT and GSK3 phosphorylation. Importantly, BDA-366 also reduced Mcl-1 levels and inhibited both basal and anti-IgM-induced phosphorylation of Bcl-2 at Ser70. The latter finding is consistent with published data^24^ and indicates that some of the reported effects of BDA-366 on Bcl-2 conformation or function may have been caused by inhibition of the signaling pathway that phosphorylates Bcl-2 rather than a direct effect on Bcl-2 (Fig. 7c).

### BDA-366 sensitizes CLL and venetoclax-resistant DLBCL cell lines to venetoclax

**Fig. 8:**
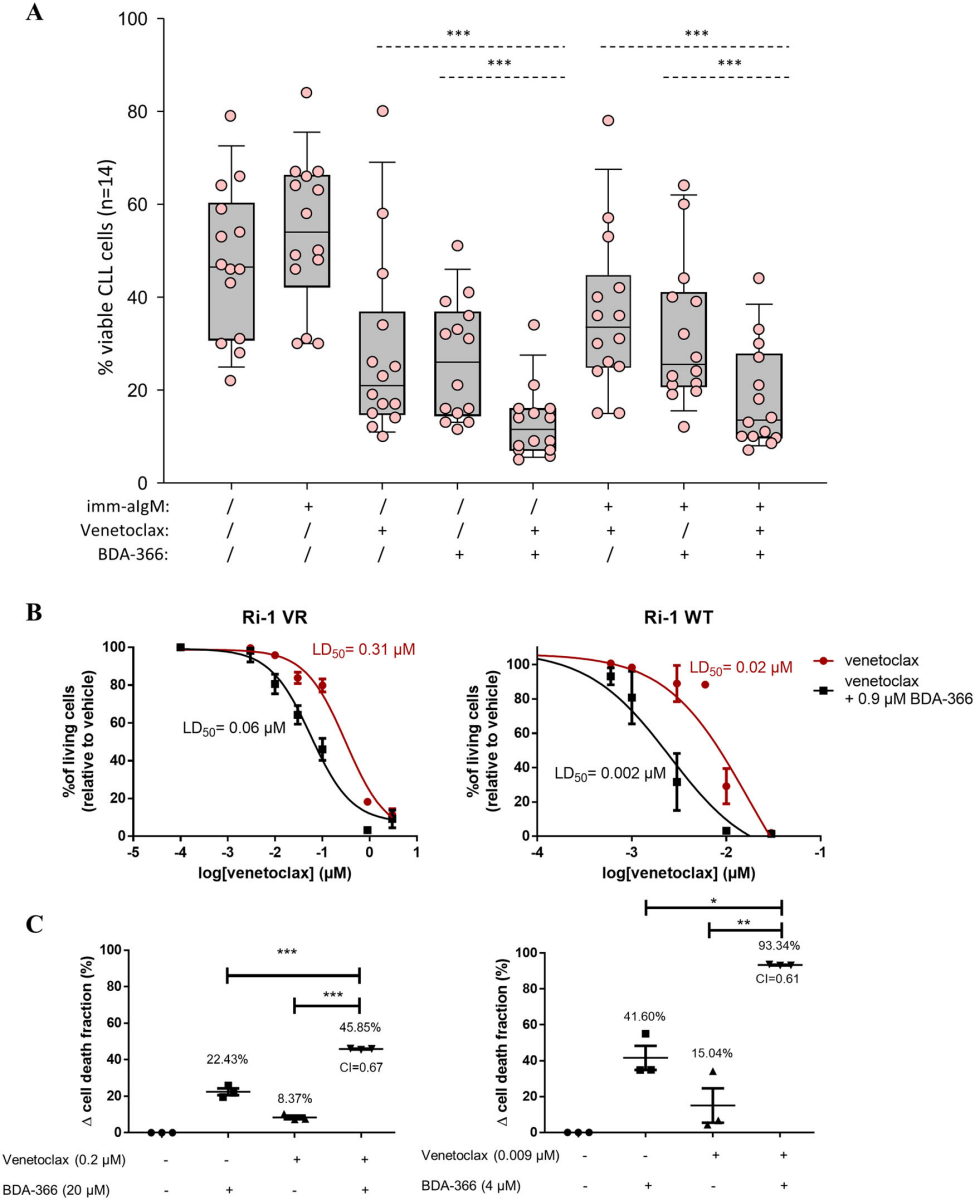
Combined treatment of BDA-366 and venetoclax sensitizes both CLL and venetoclax-resistant DLBCL cells towards venetoclax. **a** CLL cells were first cultured with or without imm-aIgM for 3 h prior to the addition of BDA-366 (1 μM) and/or Venetoclax (2 nM). Cell viability was determined after 24 h by Annexin/PI staining. Statistical analysis was done using one way repeated measures ANOVA with Holm–Sidak’s test for multiple comparisons. **b** Ri-1 (Ri-1 WT) and venetoclax resistant Ri-1 (Ri-1 VR) cells were treated with increasing concentrations of venetoclax with or without a submaximal concentration of BDA-366 (0.9 µM). Cell death was measured after 24 h using flow cytometry of Annexin V-FITC/7-AAD stained cells. Data represent average ± SEM of at least three independent experiments (*N* > 3). **c** Wild-type (Ri-1 WT) and venetoclax-resistant (Ri-1 VR) Ri-1 cells were incubated with either venetoclax, BDA-366 or a combinational treatment venetoclax + BDA-366. After 24 h the cell death was measured using flow cytometry of Annexin V-FITC/7-AAD stained cells and plotted as the treatment-induced apoptotic fraction. A combinational index (CI) was calculated as CI = (*E*_BDA-366_ + *E*_venetoclax_)/*E*_BDA-366+venetoclax_) where CI < 1 indicates synergy. Data are represented as average ± SEM of *N* > 3. Statistical significance was determined with a one-way ANOVA with a Bonferroni post hoc test comparing *E*_BDA-366_ or *E*_venetoclax_ with *E*_BDA-366+venetoclax_.

Given the observation that BDA-366 decreased Mcl-1 expression and that Mcl-1 is a key mediator of apoptosis resistance in CLL cells, we investigated viability of BDA-366-treated CLL cells following stimulation with immobilized anti-IgM, which is known to increase CLL cell survival by upregulating Mcl-1^33,34^. This increase in Mcl-1 also contributes to the resistance of CLL cells towards venetoclax, as previously shown through RNA intereference experiments^35^. Pretreatment of CLL cells with immobilized anti-IgM resulted in increased resistance to both BDA-366 and venetoclax. Interestingly, the combination of the two agents was significantly more toxic than each agent alone against anti-IgM-stimulated CLL cells (Fig. 8a), suggesting that a combination of these drugs could be effective in overcoming resistance induced by BCR signals. Similar results were obtained when venetoclax was combined with the MCL-1 inhibitor S63845, further suggesting that the venetoclax-sensitizing effect of BDA-366 in CLL was primarily mediated through MCL-1 downregulation (Supplementary Fig. 6A). Moreover, both venetoclax-sensitive (Ri-1 WT) and venetoclax-very resistant (Ri-1 VR) Ri-1 cells were treated with increasing concentrations of venetoclax and the concentration-response curves were plotted (Fig. 8b). The LD_50_ values of 0.31 and 0.02 µM reflect the difference in the sensitivity towards venetoclax in the resistant (Ri-1 VR) and WT Ri-1 cell line, respectively. Adding a fixed submaximal concentration of BDA-366 to a submaximal concentration of venetoclax resulted in a fivefold and tenfold decrease of the LD_50_ value in the wild-type and resistant Ri-1 cell line, respectively (Fig. 8b). To mathematically determine if a synergistic or additive effect exists between BDA-366 and venetoclax, we calculated the combination index (CI) (Fig. 8c). The CI was lower than 1, which is indicative for synergy. Thus, combining a submaximal concentration of BDA-366 with venetoclax consistently induced synergistic cell death in both the wild-type and resistant cell line compared to single treatment alone, which was by itself ineffective to induce cytotoxicity. A higher BDA-366 concentration was used for Ri-1 VR cells than for Ri-1 WT cells, since Ri-1 VR cells are also more resistant to BDA-366^36^. Similar results were obtained when Mcl-1 inhibitor S63845 was used instead of BDA-366 (Supplementary Fig. 6b, c). This indicates that BDA-366 holds potential to sensitize cancer cells that have become resistant towards venetoclax either through signals from the microenvironment or through prolonged exposure to the BH3 mimetic drug.

## Discussion

The non-peptidic small molecule BDA-366 has previously been described to function as a Bcl-2 antagonist by targeting Bcl-2’s BH4 domain^23^, thereby inducing apoptosis in lung cancer and multiple myeloma cells^24^. Here, we explored the potential of BDA-366 to kill Bcl-2-dependent CLL and DLBCL cells and scrutinized the mechanism of action, originally proposed to rely on a conformational switch in Bcl-2 by the drug triggering it to expose its BH3 domain and to activate Bax.

We hereby show for the first time that BDA-366 induces apoptotic death in primary CLL and DLBCL cells. Moreover, there is a therapeutic window for drug use as CLL cells appear more sensitive to BDA-366 than normal PBMCs. BMK cells lacking Bax/Bak were more resistant to BDA-366 than wild-type BMK cells, indicating that the BDA-366-induced cell death is at least in part dependent on Bax/Bak. At the mechanistic level, BDA-366 induces Bax activation in intact cells, resulting in caspase-dependent cell death. Yet, BDA-366 by itself did not directly activate Bax in an in vitro Bax-mediated liposome permeabilization assay. Moreover, our data also show that BDA-366-induced cell death is not related to Bcl-2 expression, as originally proposed. In particular, we observed that the rate of BDA-366-induced apoptosis did not correlate with Bcl-2 protein levels neither in primary CLL samples nor DLBCL cell lines. Second, overexpressing Bcl-2 in DLBCL cell lines or primary human CLL cells did not result in sensitization of the cells towards BDA-366, as one would expect if the drug functioned by converting Bcl-2 into a proapoptotic Bax-activating protein, but rather had a protective effect. Finally, BDA-366 was unable to switch Bcl-2 into a proapoptotic, Bax-activating protein in the in vitro Bax-mediated liposome permeabilization assay, further indicating that BDA-366 functions independently of Bcl-2. These findings are consistent with a recent BH3-profiling study scrutinizing a wide variety of putative Bcl-2 inhibitors using cell models with engineered addiction to distinct Bcl-2-family members^11^. BDA-366 was among the tested small molecules, but in contrast to what would be expected from a selective Bcl-2 inhibitor, BDA-366 was similarly potent in inducing cell death in Bcl-2, Bcl-xL, and Mcl-1-addicted cell models.

Our data suggest that the BDA-366-mediated downregulation of Mcl-1, caused at least in part by an increase in the Mcl-1-turnover rate, contributes to the mechanism of action of this compound. Mcl-1 is an antiapoptotic Bcl-2-family member characterized by a rapid turnover. The MCL1 gene is amplified in many human cancers resulting in increased tumor cell survival and chemotherapy resistance^37^. Another important mechanism of Mcl-1 overexpression in tumors is activation of the PI3K/AKT pathway, which increases the translation efficiency and stability of the Mcl-1 protein by activating mTORC1 and inactivating GSK3, respectively^27,32^. Previous studies by our group have shown that Mcl-1 induction through BCR-mediated activation of the PI3K/AKT pathway is an important mechanism of apoptosis resistance in CLL and DLBCL^28,33–35^. Since BDA-366 belongs to a class of compounds that are known to inhibit the PI3K/AKT pathway, we investigated how this drug affects the activity of AKT and GSK3 in human CLL cells and DLBCL cell lines. Strikingly, short duration treatment with BDA-366 resulted in reduced levels of phosphorylated and activated AKT and phosphorylated and inactivated GSK3. This effect was accompanied by downregulation of Mcl-1 and reduced phosphorylation of Bcl-2, which has been shown to enhance the antiapoptotic activity of Bcl-2 by stabilizing interactions with Bim and Bax^38,39^. Importantly, the capacity of BDA-366 to inhibit Bcl-2 Ser70 phosphorylation could potentially explain some of the discrepancies between our results and the study of Han et al.^23^ as the reported changes in Bcl-2 conformation or function may have been an indirect effect caused by inhibition of Bcl-2 Ser70 phosphorylation. This site has been reported to be phosphorylated by several different kinases, including MAP kinases, PKCalpha, PKCdelta, and GSK3^40–44^. Thus, inhibition of signaling pathways that regulate Mcl-1 expression and Bcl-2 phosphorylation may account for the Bcl-2 conformation-independent mechanisms of apoptosis that were observed in our study. Along these lines, it is also worth noting that BDA-366 was originally designed as an inhibitor of PKC^45^ and different isoforms of this enzyme have been implicated in regulating Mcl-1 expression and Bcl-2 phosphorylation^35,40,43^. Moreover, since AKT phosphorylation of Bax at residue S184 has been reported to enable Bax to bind and sequester proapoptotic BH3 proteins and prevent Bax from inserting into the mitochondria, part of the activity of BDA-366 may be derived from blocking these antiapoptotic effects^46^.

Another potential mechanism of action of BDA-366 could be related to the fact that this drug is structurally related to anthracyclines. This class of drugs has already been used in the clinic for several decades to treat cancers, such as leukemias and lymphomas^47,48^. The main modes of action of these drugs are Topoisomerase II inhibition, DNA intercalation and production of reactive oxygen species, which are all events that can potentially induce apoptosis in CLL and DLBCL cells. Interestingly, anthracyclines have been reported to provoke Mcl-1 downregulation and thereby synergizing with ABT-263, a non-selective Bcl-2/Bcl-xL inhibitor to kill cancer cells^49^. Thus, the mechanism of action of BDA-366 could be multifaceted, involving multiple pathways that cause cell damage and apoptosis.

In addition to its antiapoptotic function, Mcl-1 participates in the oxidative phosphorylation^50^. Hence, ubiquitin-dependent degradation of Mcl-1 has been shown to reduce the O_2_ consumption and ATP production and inhibited the aerobic metabolism both in pancreatic cancer and primary cell lines^51^. Moreover, deprivation of glucose resulted in Mcl-1-translational inhibition in an mTORC1-dependent manner without affecting other members of the Bcl-2 family like Bcl-2 and Bcl-xL^52^. Consequently, it seems that a mutual effect between mitochondrial metabolism and Mcl-1 translation exist. Therefore, it is possible that BDA-366 negatively affects mitochondrial metabolism thereby decreasing Mcl-1. However, further research is needed to investigate the role of BDA-366 on mitochondrial bioenergetics. Moreover, we cannot exclude a direct interaction between BDA-366 and Mcl-1 thereby priming Mcl-1 for its degradation.

The Bcl-2 inhibitor venetoclax has demonstrated considerable clinical activity in patients with various hematological malignancies^53^. Results have been particularly impressive in CLL, where an overall response rate of around 80% has been observed across all prognostic groups. However, many patients do not respond or initially respond but subsequently progress. Resistance to venetoclax appears to be primarily caused by mutations in Bcl-2 or compensatory overexpression of Mcl-1 and other antiapoptotic Bcl-2-family members^28,35,53–57^. In addition, Bcl-2 phosphorylation at Ser70 has been shown to induce a structural alteration in the BH3-binding groove that reduces by 100–300-fold the binding affinity of venetoclax^39^. In that study, the ratio of Mcl-1 + phospho-Bcl-2 over total Bcl-2 provided the most significant predictive marker for venetoclax sensitivity in a panel of CLL samples. Thus, by downregulating Mcl-1 and inhibiting Bcl-2 Ser70 phosphorylation, BDA-366 would be expected to increase the cytotoxic potential of venetoclax. Moreover, since BDA-366 decreases Mcl-1-protein levels, another possibility is that cells may shift Mcl-1-bound proapoptotic Bcl-2-family members to other antiapoptotic Bcl-2 proteins, like Bcl-2 itself. This would load Bcl-2 with proapoptotic Bcl-2 family members, thus increasing the effectiveness of venetoclax. Vice versa, by inhibiting Bcl-2, venetoclax may sensitize the malignant cells to the direct proapoptotic activities of BDA-366, similar to what has been reported for the combination of venetoclax with the anthracycline-based chemotherapy regimen R-CHOP^58^. Thus, the combination of venetoclax with BDA-366 may represent a novel potential therapeutic strategy that deserves further investigation.

## Materials and methods

### Cells

Blood samples were collected from patients who satisfied standard morphologic and immunophenotypic criteria for CLL. Patients were untreated or had not received treatment for at least 6 months prior to the study. Informed consent was obtained from all patients according to the Declaration of Helsinki, and approval for the study was obtained from the institutional human research committee at the Catholic University Hospital A. Gemelli. Mononuclear cells were isolated from peripheral blood samples by Ficoll gradient centrifugation. The proportion of CD5+ CD19+ CLL cells was >80% in all analyzed cases. CLL cells were cultured at a cell density of 1 × 10^7^/mL in RPMI 1640 supplemented with 10% heat-inactivated FBS, 100 U/mL penicillin, 0.1 mg/mL streptomycin, 2 mM l-glutamine, and 1 mM sodium pyruvate (Invitrogen). Dynabeads M-450 Epoxy 2 × 10^7^/mL (Invitrogen Dynal) coated with 20 μg/ml goat anti-human IgM (Southern Biotechnology Associates) were used for BCR cross-linking.

SU-DHL-4, KARPAS-422, PFEIFFER, TOLEDO, SU-DHL-6, OCI-LY-1, and OCI-LY-18 DLBCL cell lines were kindly provided by Dr. Anthony Letai (Dana-Farber Cancer Institute, Boston, MA, USA). The Ri-1 DLBCL cell line was ordered via DSMZ (Braunschweig, Germany). Ri-1 cells resistant to venetoclax were generated by prolonged culture of Ri-1 cells in increasing concentrations of venetoclax. All cell cultures were regularly screened for mycoplasma. These cell lines were authenticated by the University of Arizona Genetics Core (Tucson, AZ, USA) using autosomal short tandem repeat (STR) profiling via Science Exchange (www.scienceexchange.com). The results were validated using reference databases such as DSMZ (Germany) and sample profiles (allelic values) and electropherogram trace data were provided. All cell lines except one displayed a perfectly matched profile with 8 tested alleles (8/8), while for SU-DHL-6 cells 7/8 alleles matched. The SU-DHL-4, KARPAS-422, PFEIFFER, TOLEDO, Ri-1, SU-DHL-6, HT, HT overexpressing Bcl-2 (HT Bcl-2), Ri-1 and venetoclax-resistant Ri-1 DLBCL cell lines were cultured in suspension in RPMI-1640 media. The OCI-LY-1 and OCI-LY-18 DLBCL cell lines were cultured in suspension in Iscove modified Dulbecco medium (Invitrogen, Merelbeke, Belgium). All media were supplemented with 10% heat-inactivated fetal bovine serum (FBS), l-glutamine (100× GlutaMAX, Gibco/Invitrogen, Merelbeke, Belgium) and penicillin and streptomycin (100× Pen/Strep, Gibco/Invitrogen, Merelbeke, Belgium) and cultured at 37 °C and 5% CO_2_. Wehi7.2 and Wehi7.2 overexpressing Bcl-2 (Wehi7.2 Bcl-2) were cultured in 10% DMEM + G418 (1000 μg/ml) at 37 °C and 10% CO_2_. Baby mouse kidney (BMK) cells and their *Bax*^*−/−*^ *Bak*^*−/−*^ derivatives were gifts from Eileen White (Rutgers University, Piscataway, NJ, USA). BMK cells were cultured in DMEM containing 10% FBS.

### Antibodies and reagents

Small molecule BDA-366 was received from the Drug Synthesis and Chemistry Branch, Developmental Therapeutic Program, Division of Cancer Treatment and Diagnosis, National Cancer Institute (NCI, Bethesda, MD). AffiniPureF(ab’)2FragmentGoatAnti-HumanIgG + IgM (H + L) (IgG/IgM) was purchased from SANBIO (CA, USA).

Immunoblotting was performed on DLBCL with anti-vinculin (#V-9131,Sigma-Aldrich, Munich, Germany), anti-GAPDH (#G8795, Sigma-Aldrich, Munich, Germany), anti-Bax (6A7) (#Ab00120-1.1, Absolute Antibody, Oxford, United Kingdom), anti-Bcl-2 (#PA5-20068, Thermo Scientific, Brussels, Belgium), anti-Bax (#2772, Cell Signaling, MA, USA), anti-Mcl-1 (# 4572, Cell Signaling, MA, USA).

Immunoblotting of primary CLL patient samples was performed using the following antibodies: anti-PARP (#9542, Cell Signaling), anti-phospho-Bcl2 (Ser70) (#2827, Cell Signaling), anti-phospho AKT (Ser473) (#9271, Cell Signaling), anti-phospho-GSK3α/β (Ser21/9) (#9331, Cell Signaling), anti-Bim (#2819, Cell Signaling), anti-Bcl-2 (#2872, Cell Signaling), anti-β-actin (#3700, Cell Signaling), anti-Mcl-1 (#sc-819 Santa Cruz Biotechnology), anti-Bcl-xL (#sc-1041, Santa Cruz Biotechnology), anti-Bax (#sc-493, Santa Cruz Biotechnology), rabbit IgG-HRP-linked (# 111-035-046, Jackson ImmunoResearch), and mouse IgG-HRP-linked (#115-035-07, Jackson ImmunoResearch).

### Western blotting

Cells were washed with phosphate-buffered saline and incubated at 4 °C with lysis buffer (20 mM Tris-HCl (pH 7.5), 150 mM NaCl, 1.5 mM MgCl_2_, 0.5 mM dithiothreitol, 1% Triton X-100, 1 tablet complete EDTA-free protease inhibitor per 50 ml (Thermo Scientific, Brussels, Belgium) for 30 min on a head-over-head rotor. Cell lysates were centrifuged for 5 min at 10,000 rpm. Totally, 10–20 µg of protein sample was loaded on a NuPAGE 4–12% Bis–Tris protein gel (Life Technologies, Brussels, Belgium) and analyzed by western blotting. Immunodetection and quantification were done on an ALLIANCE LD2 chemiluminescence Imaging System (Cleaver Scientific Ltd., Warwickshire, UK), using ECL Plus enhanced-chemiluminescence detection reagents (GE Healthcare, Chicago, IL, USA).

### Immunocytochemistry and co-IP to detect active Bax

The procedure for immunocytochemistry was described previously^59^. Briefly, cells were plated at 500,000 cells/ml on a poly-l-lysine coated plate and treated with 20 µM BDA-366 or vehicle control. After 6 h of incubation, cells were washed once quickly with ice-cold phosphate-buffered saline (PBS) and fixed with 2% PFA for 15 min at room temperature. Afterwards, cells were washed twice with ice-cold PBS supplemented with 2% bovine serum albumin (BSA) and permeabilized for 15 min with 0.2% Triton-X-100 in PBS containing 2% BSA. Cells were then washed twice with ice-cold 2% BSA containing PBS and blocked with 10% goat serum for 30 min at room temperature. Following the blocking, cells were loaded with the primary antibody (1.3 µg anti-Bax (6A7) in 2% BSA containing PBS) overnight at 4 °C. After two additional washes the cells were incubated with 1:175 Alexa 488 goat anti-mouse IgG secondary antibody for 1 h at room temperature. Cells were washed twice and stained for 2 min with 10 µg/ml Hoechst 33342 to detect the apoptotic nuclei. Images were taken using Zeiss Axio Observer Z1 Inverted Microscope equipped with a ×20 air objective and a high-speed digital camera (Axiocam Hsm, Zeiss, Jena, Germany).

For the co-immunoprecipitation experiments cells were lysed in a CHAPS-based lysis buffer (50 mM Tris-HCl pH 7.5, 100 mM NaCl, 2 mM EDTA, 50 mM NaF, 1 mM Na_3_VO_4_, 1% CHAPS and protease inhibitor tablets (Roche, Basel, Switzerland)). Totally, 50 µg of cell lysate was incubated with 5 µg antibody (anti-Bax (6A7)/mouse IgG) in lysis buffer for 2 h at 4 °C using a head-over-head rotor. After washing the Pierce protein A/G magnetic beads two times with washing buffer (0.05% Tween20 and 0.5 M NaCl in TBS), they were added to the lysate-antibody mixture and incubated overnight at 4 °C. Samples were washed 3 times with lysis buffer and boiled for 5 min at 95 °C in 30 µl of 2× LDS sample buffer containing 1:200 β-mercaptoethanol. The beads were manually removed from the solution using a magnetic stand.

### Apoptosis assay

Totally, 5 × 10^5^ cells/ml DL-BCL cells were treated for 24 h with increasing concentrations of either BDA-366/venetoclax or a single concentration of venetoclax/BDA-366 or the combination of both treatments. Afterwards, cells were pelleted by centrifugation, and incubated with Annexin V-FITC (Life Technologies, Brussels, Belgium) and 7-aminoactinomycin D (7-AAD) (Life Technologies, Brussels, Belgium) or with 2.5 µM NucviewTM 488 caspase-3 substrate (Biotium, CA, USA) for 15 or 30 min, respectively. Cell suspensions were analyzed with an Attune^®^ Acoustic Focusing Flow Cytometer (Applied Biosystems, Brussels, Belgium). Cell death was scored by quantifying the population of Annexin V-FITC-positive and 7-AAD positive cells or by quantifying the caspase-3 positive cells. After treating the BMK cells (3000 cells/well in 384-well plate) for 24 h with BDA-366, cells were stained for 30 min with DRAQ5, TMRE and Annexin V-Alexa fluor 488. Images were taken using the Opera High Content Screen System (PerkinElmer) with ×20 air objective. Intensity and morphology features were extracted from the fluorescent images through image segmentation and analyzed using Acapella analysis software (PerkinElmer) script (available for free at http://www.andrewslab.ca). Quantitative analyses of cell death results in cell lines are from cell populations in which at least 80% of the cells were viable in control conditions.

### Liposome permeabilization assay

The liposome permeabilization assay was performed as previously described^60^. Briefly, unilamellar liposomes (48% phosphatidylcholine, 28% phosphatidylethanolamine, 10% phosphatidylinositol, 10% dioleoyl phosphatidylserine, and 4% tetraoleoyl cardiolipin (Avanti)) were encapsulated with the quencher, p-xylene-bis-pyridinium (DPX, 45 mM, Life Technologies) and the fluorescent dye, aminonaphthalene1,3,6-trisulfonic acid (ANTS, 12.5 mM, Life Technologies) in assay buffer (10 mM Hepes (pH 7.2), 200 mM KCl, 1 mM MgCl_2_).

The release of encapsulated ANTS dye (excitation at 355 nm and emission at 520 nm) from liposomes (1 mg/mL) was measured on a Tecan M1000 microplate reader to assess activation of Bax via Bax-mediated pore formation. Background (*F*_0_) was recorded for 30 min at 37 °C in the presence of 0.04 mg/mL liposomes and the indicated compounds and proteins (DMSO [1%], BDA-366, ABT-199, Bcl-xL, Bcl-2^ΔTMD^ and Bim or cBid). Afterwards Bax was added (*t*_0_) and change in fluorescence (*F*) was examined for 2 or 3 h at 37 °C. Finally, the liposomes were permeabilized with Triton-X-100 (0.2%) and fluorescence was measured during 5 min to estimate F100. The % release is calculated as follows: [(*F* − *F*_0_)/(*F*_100_ − *F*_0_)] × 100%.

### Protein purification

Recombinant Bax, Bim, cBid, and Bcl-xL were expressed and purified as described^60^. For the purification of recombinant Bcl-2, *Escherichia coli* are transformed (BL21-AI) with an amino terminal 6× histidine-tagged Bcl-2^ΔTMD^-expressing plasmid (pET47‐Bcl‐2^∆23^) and plated on LB-kanamycin agar. The next day a colony was picked for overnight growth at 37 °C with shaking in DYT medium + kanamycin. Afterwards the overnight culture was mixed with DYT medium + kanamycin and grown at 37 °C with shaking when the bacterial growth is in log phase (OD_600_ = 0.2) the culture was heat-shocked for 2 h at 40 °C. At this point protein expression is induced with IPTG (100 µM) for 2 h at 20 °C. Bacteria were harvested by centrifugation, resuspended in lysis buffer (150 mM NaCl, 10 mM Tris pH 7.4, 20% glycerol, 30 mM Imidazole) and lysed by sonication. Lysed cells were centrifuged (35,000 rpm for 40 min) and supernatants were incubated with NiNTA beads (Thermo Fisher Scientific) for 1 h at 4 °C while rotating. Bcl-2^ΔTMD^ is eluted with lysis buffer containing increasing concentrations of Imidazole (4 fractions with respectively 30 mM, 100 mM, 250 mM and 500 mM Imidazole in lysis buffer).

### Measurement of the Mcl-1 turnover

HT and HT Bcl-2 cells were incubated with 20 µg/ml cycloheximide (CHX) (Sigma Aldrich, Brussels, Belgium) in combination with 4 µM Ac-DEVD-CHO (Biotium, CA, USA) to inhibit caspase-3. In addition, the proteasome inhibitor MG-132 (20 µM) was used for 4 h in the presence of CHX and the caspase-3 inhibitor. Samples for immunoblotting were taken at the indicated time points.

### CD experiments

CD spectra were recorded using a Jasco J-1500 spectropolarimeter (Oklahoma City, OK, USA) equipped with a Peltier element for temperature control and a six-position cuvette holder. Proteins were dialyzed in 5 mM MOPS pH:7.5; 5 mM NaCl, for 15 h, at 4 °C; 3 changes; constant stirring. Aggregated material was removed by centrifugation (20,000 g; 15 min; 4 °C) before protein concentration was determined on a Nanodrop instrument (280 nm; 2000 series; Thermo). The molecular extinction coefficient and molecular weight for A280 analysis was determined using the Expasy server (http://web.expasy.org/protparam/). Variable temperature measurements (15–85 °C) were performed using 15 µM protein, in 5 mM MOPS pH 7.5, 5 mM NaCl, 0.5%DMSO, in the presence or not of BDA-366 (50 µM) or ABT-737 (5 µM) (as indicated); 1 mm quartz cuvettes (Hellma, Mullheim, Germany); interval 0.5 °C; gradient 1 °C/min; DIT: 0.5 s; bandwidth: 1 nm. Data were analyzed using the SPECTRA ANALYSIS v.2 software (Jasco). Tmapp were derived by acquiring the first derivatives of the melting curves, using the calculus function of ORIGIN7 (GE).

### Ca^2+^ measurements in cell populations

To perform Ca^2+^ measurements in intact cells, SU-DHL-4 and OCI-LY-1 cells were seeded in poly-l-lysine-coated 96-well plates (Greiner) at a density of 5 × 10^5^ cells/ml. The cells were loaded for 30 min with 1.25 µM Fura-2 AM at 25 °C in modified Krebs solution, followed by a 30 min de-esterification step in the absence of Fura-2 AM. Fluorescence was monitored on a FlexStation 3 microplate reader (Molecular Devices, Sunnyvale, CA, USA) by alternately exciting the Ca^2+^ indicator at 340 and 380 nm and collecting emitted fluorescence at 510 nm. BDA-366 (3–30 µM), IgG 12 µg/ml, EGTA (final concentration 3 mM), TG (final concentration 1 µM), Ionomycin 10 µM in CaCl_2_ (final concentration 10 mM) were added as indicated. All traces are shown as the ratio of emitted fluorescence of Fura-2 (F340/F380). At least three independent experiments were performed.

### Single-cell Ca^2+^ imaging

A Zeiss Axio Observer Z1 Inverted Microscope equipped with a 20× air objective and a high-speed digital camera (Axiocam Hsm, Zeiss, Jena, Germany) were used for these measurements. Fura-2 AM measurements were performed by ratiometric excitation imaging similar to the measurements for populations, above.

### Statistical analysis

Results from the western blot analysis are expressed as average ± SD whereby *N* refers to the number of independent experiments. Correlation of the different LD_50_ values with each other and with the protein expression levels were statistically analyzed via linear regression using the GraphPad Prism Software 8.4 (or later). The statistical difference between LC50 values in wild-type versus DKO BMK cells was determined using a non-parametric Mann-Whitney test. The half-life value of Mcl-1 in HT and HT overexpressing Bcl-2 was measured by nonlinear regression analysis using the GraphPad Prism Software. To determine the synergistic effect of venetoclax in combination with BDA-366 or S63845 compared to single-compound treatments, the CI was calculated by making the ratio of the theoretical sum of the individual effects (*E*_BDA-366_ + *E*_venetoclax_ or *E*_63845_ + *E*_venetoclax_) with the effect of combining the treatments (*E*_BDA-366+venetoclax_ or *E*_S63845+venetoclax_. Statistical significance was determined with a one-way ANOVA with a Bonferroni post hoc test comparing *E*_BDA-366/S63845_ or *E*_venetoclax_ with *E*_BDA-366+venetoclax_. Statistical differences were indicated as follows: *p < 0.05, **p < 0.01, ***p < 0.001, ****p < 0.0001.

## Supplementary information

Supplemental Figure legends

Supplemental Figure 1

Supplemental Figure 2

Supplemental Figure 3

Supplemental Figure 4

Supplemental Figure 5

Supplemental Figure 6
