# Supplementary material for: BDA-366, a putative Bcl-2 BH4 domain antagonist, induces apoptosis independently of Bcl-2 in a variety of cancer cell models
Source: Cell Death Dis. 2020 Sep 17;11(9):769. doi: 10.1038/s41419-020-02944-6 (PMC7498462; doi:10.1038/s41419-020-02944-6)
Supplement: Supplementary file 1 — Supplemental Figure legends [file 41419_2020_2944_MOESM1_ESM.docx]

**Supplemental Figure Legends**

**Supplementary Figure 1. Immunoblotting analysis of cellular extracts from CLL and normal peripheral blood mononuclear cells**. The Bcl-2 positive DLBCL cell line SU-DHL4 and the Bcl-2 negative cell line OCI-LY3 were used as internal standards.

**Supplementary Figure 2. Induction of apoptosis by BDA-366 in DLBCL using a caspase-3 probe. (A)** Concentration-response curves of 12 different DLBCL cell lines and two murine thymocytes respectively: SU-DHL-4, HT, KARPAS-422, SU-DHL-6, TOLEDO, HT overexpressing Bcl-2 (HT Bcl-2), OCI-LY-1, Ri-1, PFEIFFER, OCI-LY-18, Wehi7.2 and Wehi7.2 overexpressing Bcl-2 (Wehi7.2 Bcl-2) cell lines were treated with increasing concentrations of BDA-366. After 24 hours the level of apoptosis was measured using flow cytometeric analysis after 30 minutes incubation with NucView caspase-3. Data represented here are averages ± SEM of at least 3 independent experiment (N>3). **(B)** Representative western blot of the Bcl-2 in TOLEDO, Ri-1, OCI-LY-1, OCI-LY-18, SU-DHL-6, Bcl-2 overexpressing HT (HT Bcl-2), SU-DHL-4, Bcl-2-overexpressing Wehi7.2 (Wehi7.2 Bcl-2), KARPAS-422, PFEIFFER, Wehi7.2 and HT cell lines.

**Supplementary Figure 3. Overexpression of Bcl-2 in primary human CLL cells by Bcl-2 mRNA transfection increases resistance to BDA-366. (A)** Immunoblotting analysis of CLL cells transfected with control or Bcl-2 mRNA. **(B)** Annexin V/PI analysis of Bcl-2 transfected and non-transfected CLL cells culture in the absence or presence of BDA-366 (1 μM) or venetoclax (4 nM).

**Supplementary Figure 4. Liposome permeabilisation assay in the presence of cBid. (A)** Representative plot of the liposome permeabilisation assay with BDA-366 at 0.1 and 1 μM and different Bcl-2-family members with Bax (100 nM), Bim (10 nM), Bcl-xL(40 nM). Same experimental setup as in Figure 4A. **(B)** Mean ± SD plot of the liposome permeabilisation assay with BDA-366 or ABT-199 (at 0.156 and 1.25 µM) and different Bcl-2-family members with Bax (100 nM), Bim (20 nM), Bcl-2 (100 nM). Same experimental setup as in Figure 4E. **(C)** Representative plot of the liposome permeabilisation assay with BDA-366 at 0.1, 1 and 1 μM and different Bcl-2-family members with Bax (100 nM), cBid (20 nM) and Bcl-xL (40 nM). Same experimental setup as in Figure 4A. **(D)** Data of endpoint measurements represented as averages ± SD of 4 independent experiments.

**Supplementary Figure 5.** Representation of the different independent experiments for cytosolic Ca^2+^ measurements in Fura-2 AM-loaded SU-DHL-4 and OCI-LY-1 cells. The first arrow indicates the addition of compound, being DMSO (grey curve), IgG (red curve) or BDA-366 (other curves). Experimental setup is similar to Figure 5A.

**Supplementary Figure 6. Combined treatment of venetoclax and Mcl-1 inhibitor (****S63845) sensitizes Ri-1 venetoclax-resistant cells towards venetoclax.** (A) CLL cells were first cultured with or without imm-aIgM for 3 hours prior to the addition of S63845 (100 nM) and/or Venetoclax (2 nM). Cell viability was determined after 24 hours by Annexin/PI staining. Statistical analysis was done using One Way Repeated Measures ANOVA with Holm-Sidak's test for multiple comparisons. (B) Ri-1 (Ri-1 WT) and venetoclax resistant Ri-1 (Ri 1 VR) cells were treated with S63845 (1 µM) with or without a submaximal concentration of venetoclax (0.003-0.1 µM). Cell death was measured after 24 hours using flow cytometry of Annexin V-FITC/7-AAD stained cells. Data represent average ± SD of 3 independent experiments (N=3). Statistical significance was determined with a one-way ANOVA with a Bonferroni post-hoc test. (C) Quantification of treatment-induced cell death fraction derived from B. A combinational index (CI) was calculated as CI= (E_S63845_+ E_venetoclax_)/E_S63845+venetoclax_) where CI<1 indicates synergy. Data are represented as average ± SD of N=3. Statistical significance was determined with a one-way ANOVA with a Bonferroni post-hoc test comparing E_S63845_ or E_venetoclax_ with E_S63845+venetoclax_.
