# Supplementary figures and images for: BDA-366, a putative Bcl-2 BH4 domain antagonist, induces apoptosis independently of Bcl-2 in a variety of cancer cell models
Source: Cell Death Dis. 2020 Sep 17;11(9):769. doi: 10.1038/s41419-020-02944-6 (PMC7498462; doi:10.1038/s41419-020-02944-6)

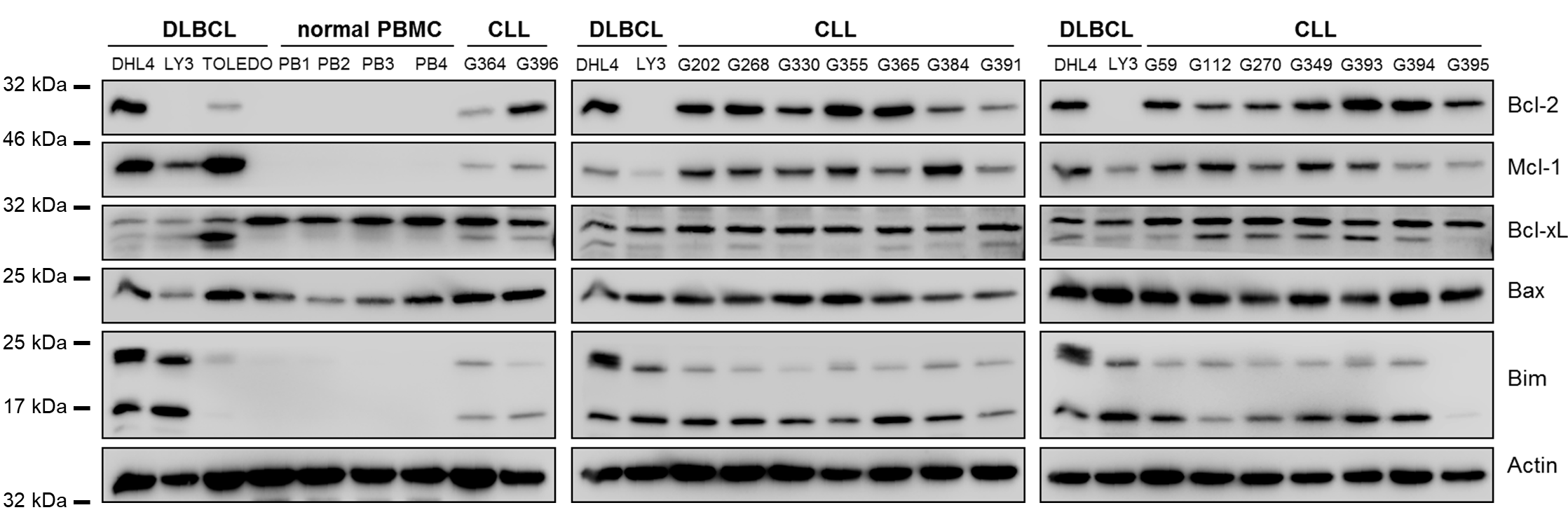

Supplement: Supplementary file 2 — Supplemental Figure 1 [file 41419_2020_2944_MOESM2_ESM.tif]

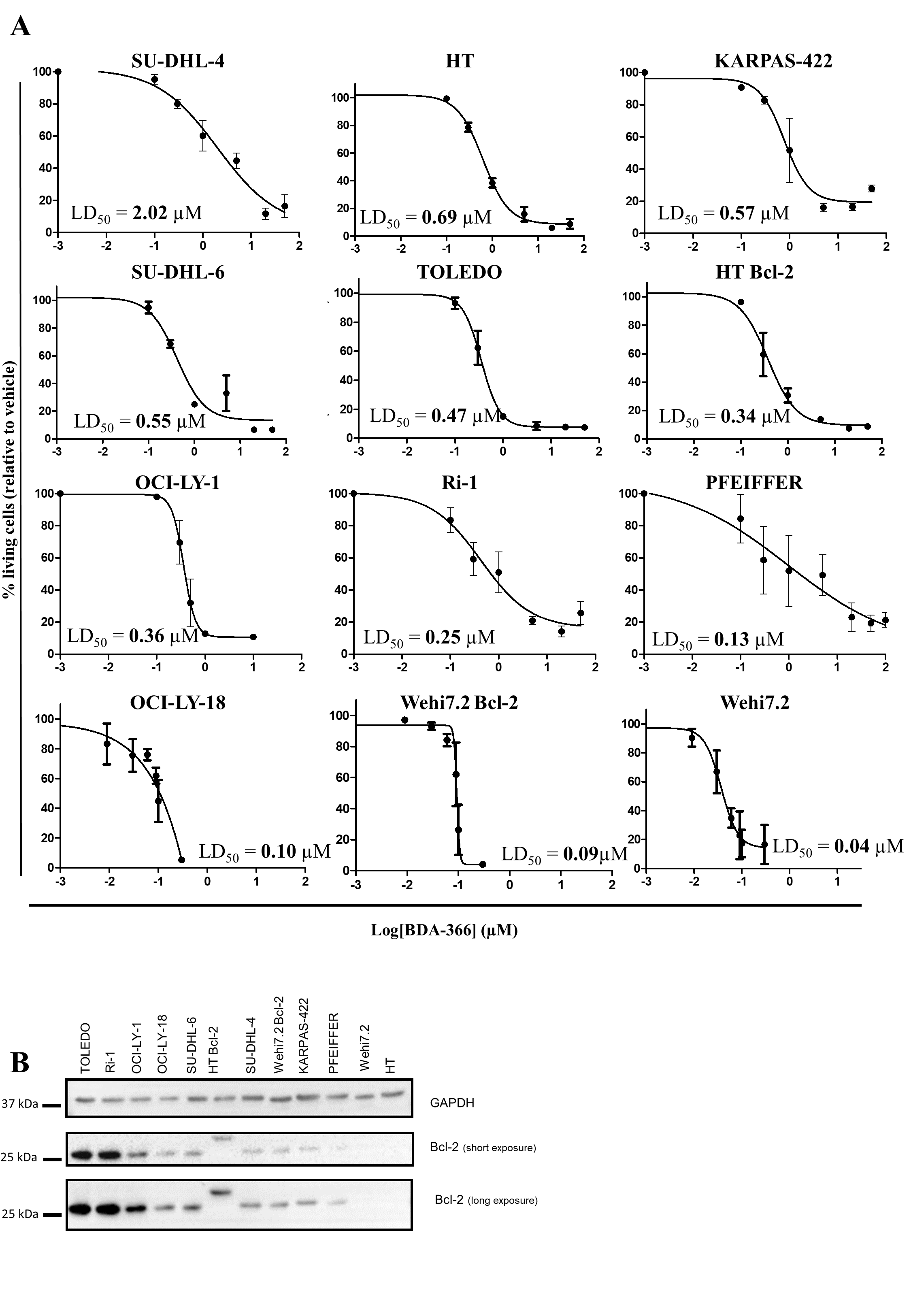

Supplement: Supplementary file 3 — Supplemental Figure 2 [file 41419_2020_2944_MOESM3_ESM.tif]

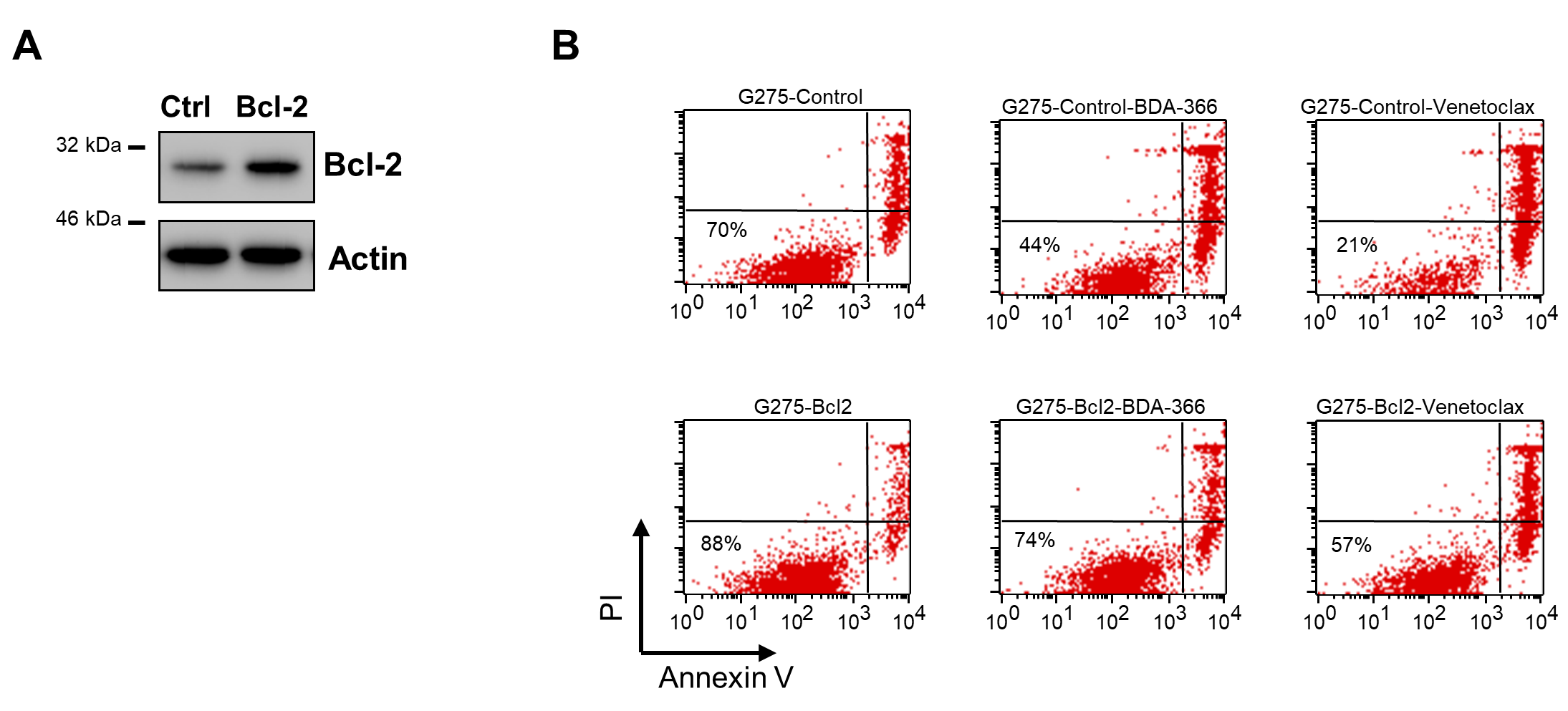

Supplement: Supplementary file 4 — Supplemental Figure 3 [file 41419_2020_2944_MOESM4_ESM.tif]

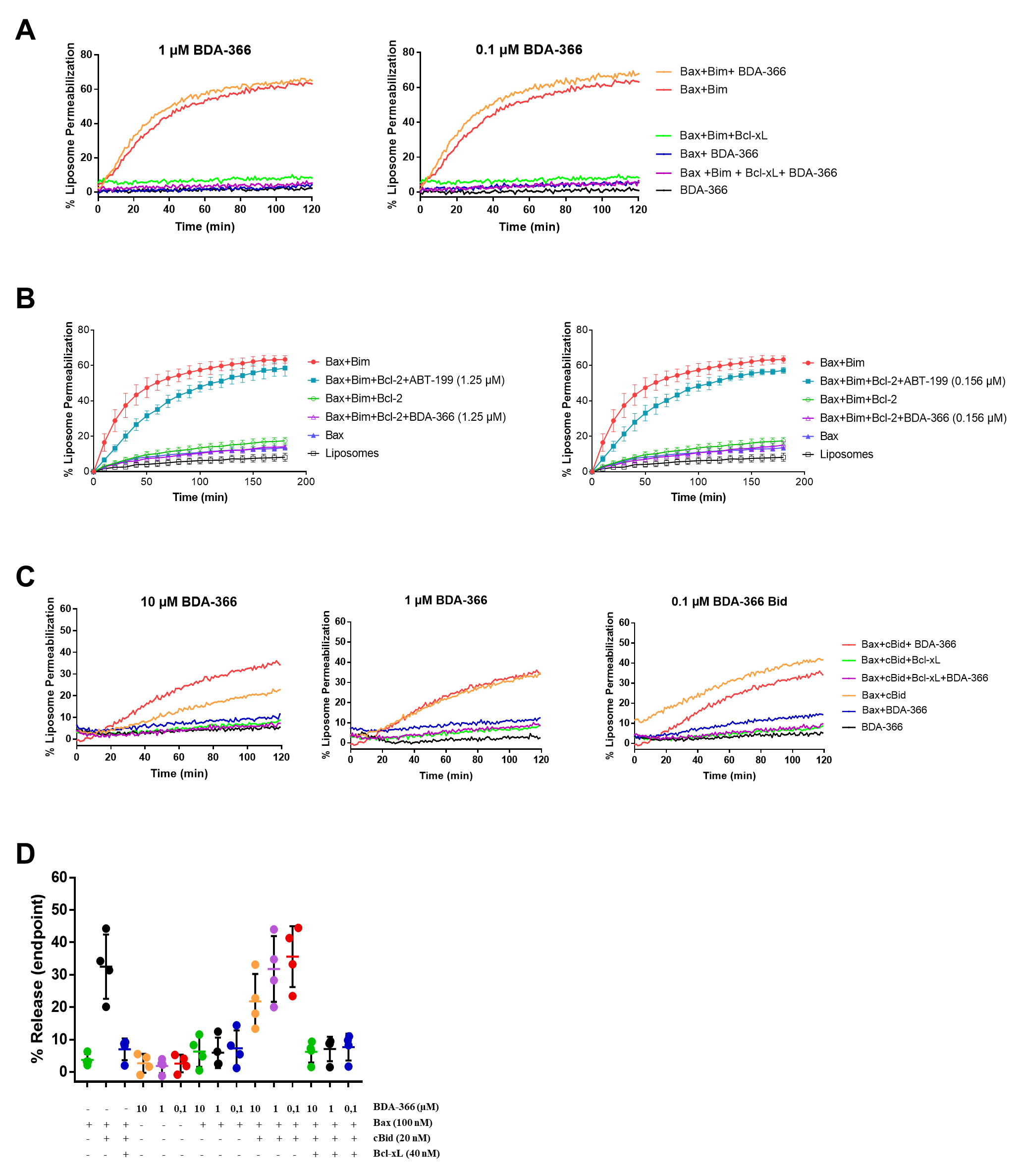

Supplement: Supplementary file 5 — Supplemental Figure 4 [file 41419_2020_2944_MOESM5_ESM.tif]

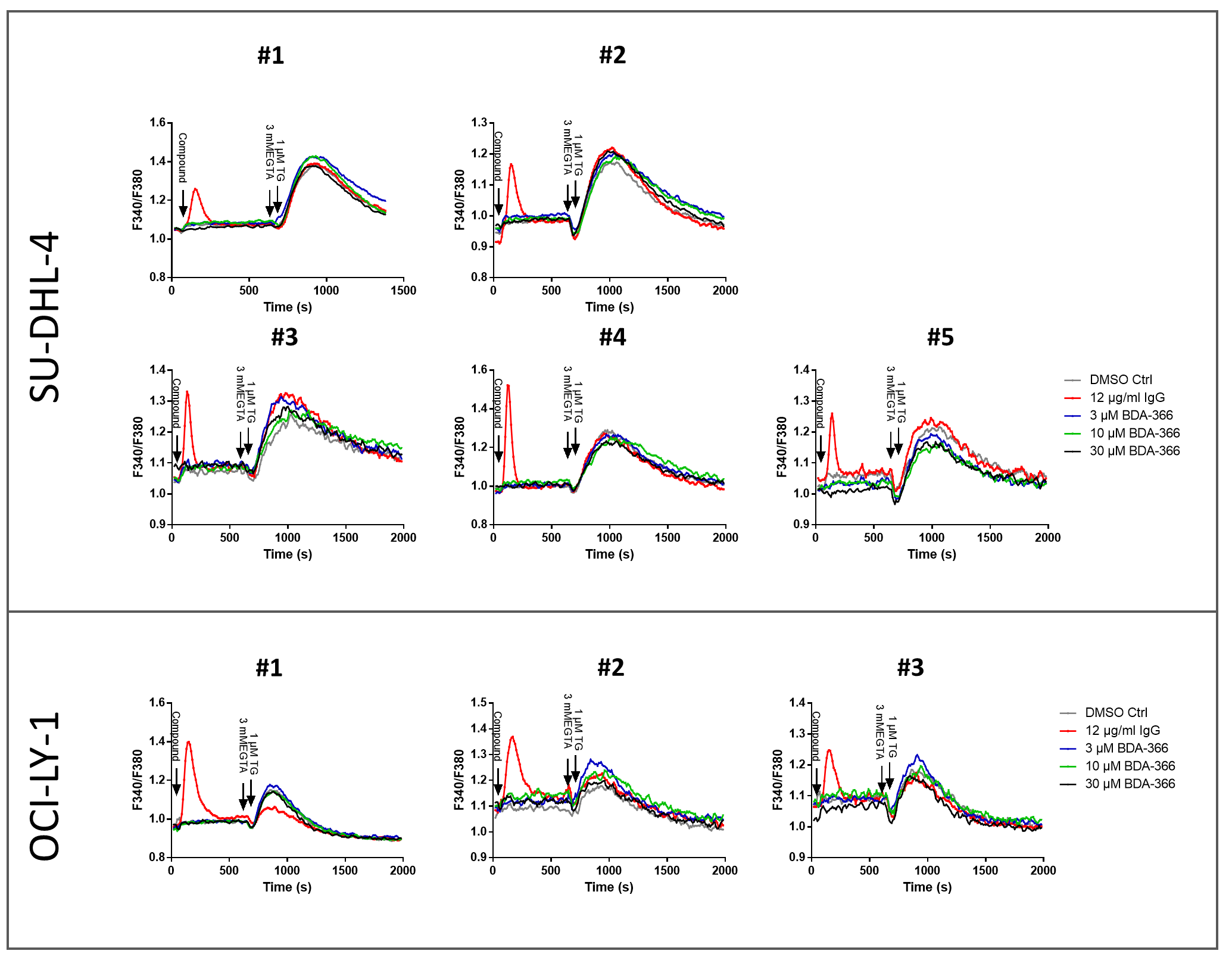

Supplement: Supplementary file 6 — Supplemental Figure 5 [file 41419_2020_2944_MOESM6_ESM.tif]

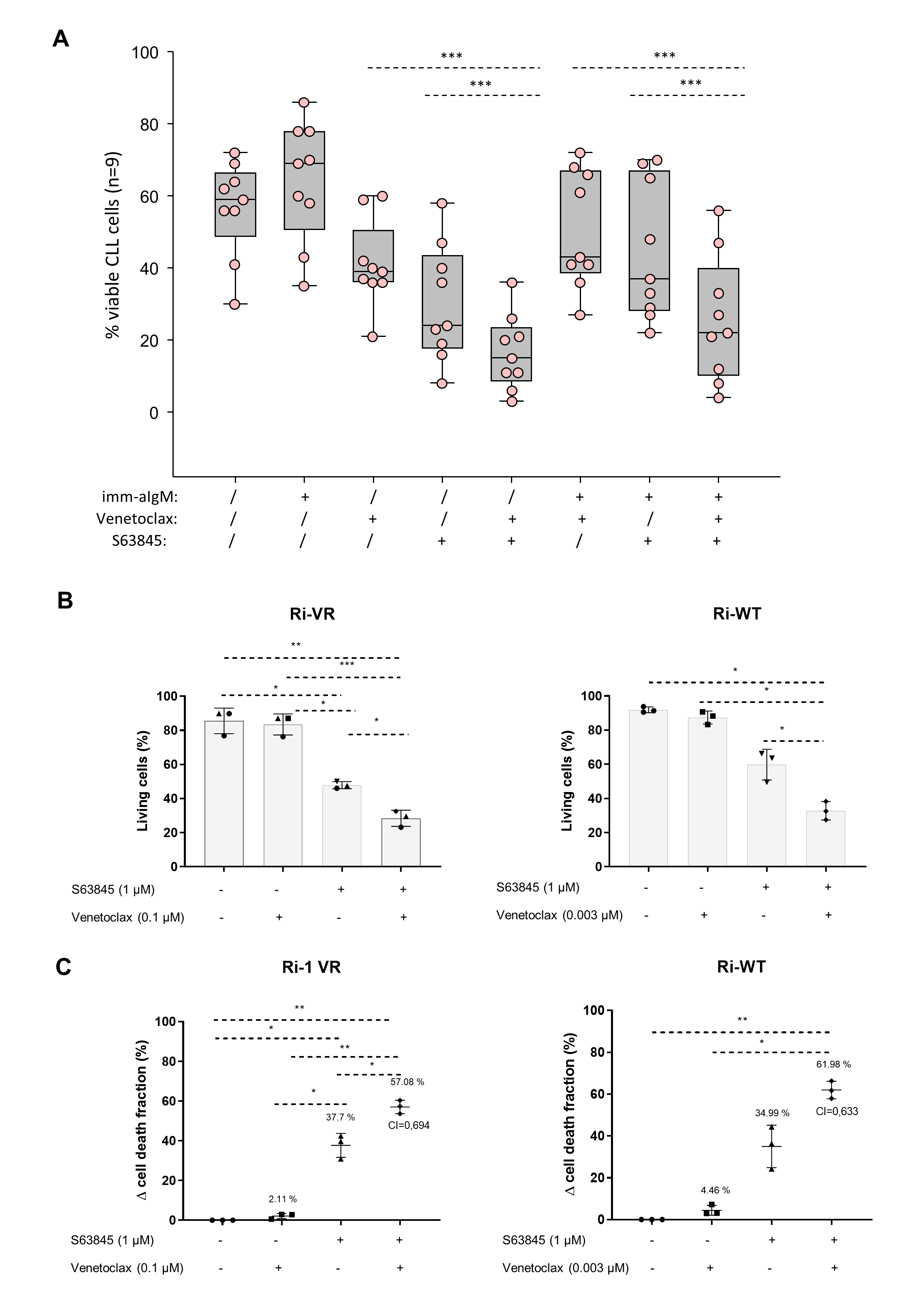

Supplement: Supplementary file 7 — Supplemental Figure 6 [file 41419_2020_2944_MOESM7_ESM.tif]
